# Methylcyclohexane-assisted polypropylene upcycling over PtRu/Al_2_O_3_*via* tandem dehydrogenation–hydrogenolysis

**DOI:** 10.1039/d6ee05130h

**Published:** 2026-10-27

**Authors:** Haokun Wang, Zhiyuan Zong, Mingyu Luo, Frederick Z. T. Yang, Jiaying Pan, Zeyu Wu, Rui Liu, Yuhong Luo, Jiasi Li, Yiyang Li, Roland C. Turnell-Ritson, Michail Stamatakis, Aidong Yang, Dermot O’ Hare

**Affiliations:** a Chemistry Research Laboratory, Department of Chemistry, University of Oxford 12 Mansfield Road Oxford OX1 3TA UK dermot.ohare@chem.ox.ac.uk; b Department of Engineering Science, University of Oxford Parks Road Oxford OX1 3PJ UK; c Inorganic Chemistry Laboratory, University of Oxford South Parks Road Oxford OX1 3QR UK

## Abstract

Catalytic hydroconversion of polyolefin waste typically relies on the addition of high-pressure hydrogen, which greatly impacts both the process implementation and economic viability. Here we report polypropylene (PP) hydrogenolysis coupled to methylcyclohexane (MCH) dehydrogenation providing an external-H_2_-free tandem route to naphtha-range liquid hydrocarbons. The best performing catalysis system was 1 nm sized PtRu nanoparticles (1 wt% Pt/1 wt% Ru) on γ-Al_2_O_3_. At 300 °C, this catalyst could facilitate over 96 wt% PP conversion with more than 88 wt% as naphtha-range liquid hydrocarbon products. This performance was retained over 6 recycles. Spectroscopic characterisation indicates the formation of electronically coupled PtRu domains, which promote MCH dehydrogenation and moderate C–C bond cleavage. Solvent screening shows that hydrocarbon liquids with different structures lead to pronounced differences in PP conversion and product selectivity. Molecular dynamics simulations of PP in representative solvents provide a qualitative picture of how solvent-dependent chain mobility and chain extension may influence polymer accessibility to catalytic sites. Techno-economic analysis and life-cycle assessment suggest that, under favorable feedstock assumptions, renewable MCH could lower both carbon intensity and production cost relative to a direct H_2_-based route. These results identify MCH-assisted PtRu nanoparticle catalysis as a viable tandem process for PP upcycling and highlight the importance of coupling hydrogen-donor chemistry with liquid-phase transport effects in plastic waste valorisation.

## Abstract 2

Polymer–solvent interactions influence polypropylene chain conformation and catalytic performance. Among the solvents tested, methylcyclohexane enhances polypropylene conversion, favouring naphtha-range liquids over gaseous products.

## Graphical abstract

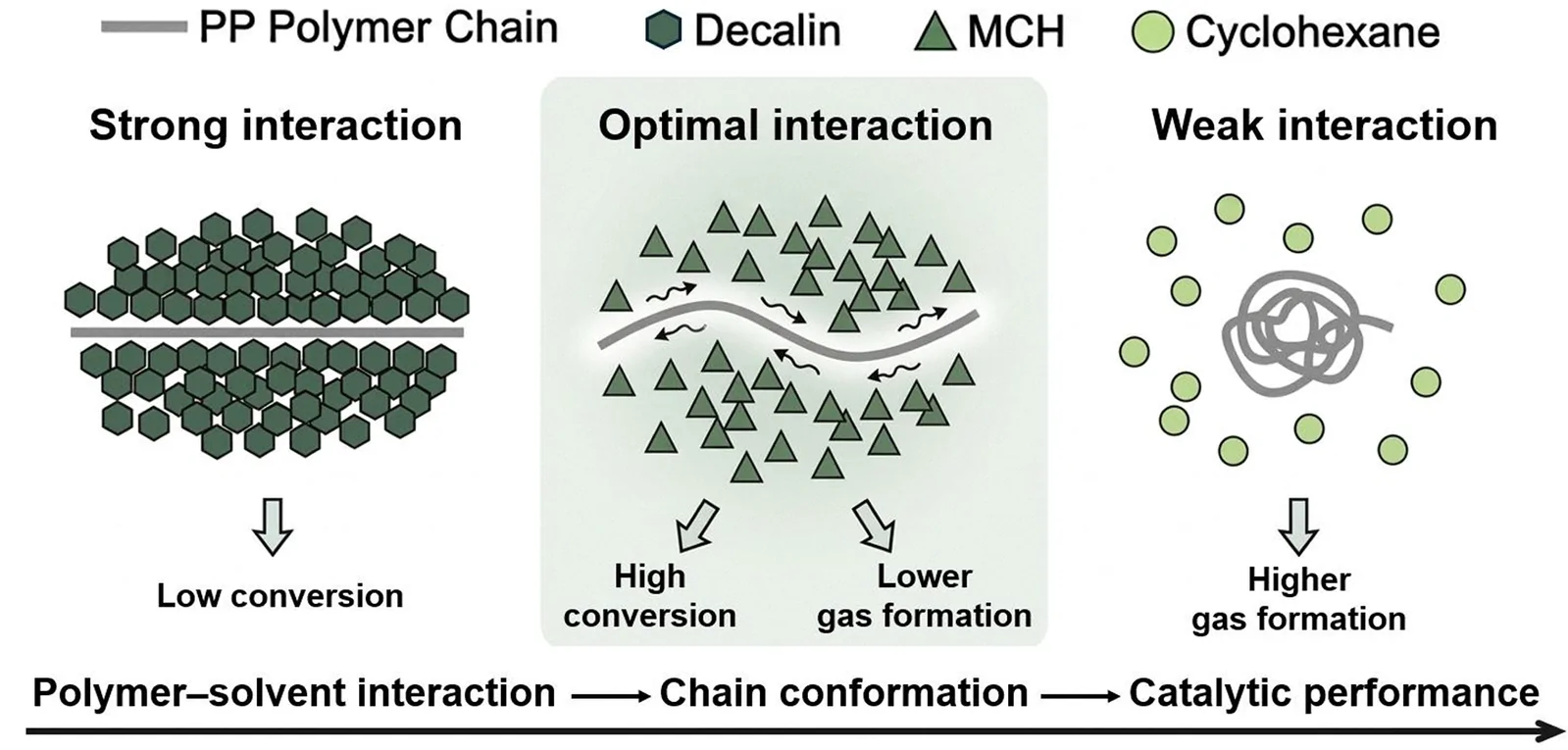

**Broader context**

Upcycling polyolefin plastic waste with liquid organic hydrogen carriers as substitutes for external hydrogen is attractive, but remains challenging because hydrogen supply, catalyst function, and liquid-phase transport are tightly coupled. Here, we show that methylcyclohexane (MCH) can serve simultaneously as a hydrogen carrier and reaction medium using a supported PtRu nanoparticle catalyst. Our catalysed tandem hydrogenolysis coupled to MCH dehydrogenation process, affords high yields of naphtha-range liquid hydrocarbon products from both virgin and consumer sourced PP. Solvent screening and molecular dynamics simulations suggest that the liquid medium influences polymer conformation and access to catalytic sites, while techno-economic and life-cycle analyses indicate that renewable MCH could offer favorable process economics and a lower carbon intensity. Collectively, these results identify liquid-phase design as a key lever in catalytic plastic upcycling, beyond hydrogen supply alone.

## Introduction

Since the advent of large-scale production in the mid-20th century, plastics have become indispensable across modern manufacturing and infrastructure owing to their low cost, light weight, durability, and chemical resistance.^1–4^ In 2024, global plastics production exceeded 430 Mt and is projected to surpass 1200 Mt annually by 2050.^5^ However, their chemical robustness also underlies their environmental persistence, leading to accumulation in landfills and natural systems with significant consequences for resource efficiency and ecosystem health.^2,6–9^ Current end-of-life treatment strategies, including incineration and mechanical recycling, remain limited by high energy demand, low-value outputs, and associated with greenhouse gas and pollutant emissions.^10–12^ In contrast, catalytic hydroconversion offers a promising route to transform plastic waste into fuels, lubricants, and value-added chemical feedstocks under relatively mild conditions (200–350 °C).^3,13–15^ Significant advances have been achieved for polyolefin (PO) plastics, including low-density polyethylene (LDPE), high-density polyethylene (HDPE), and polypropylene (PP), which account for approximately half of global plastic production and more than 60% of post-consumer plastic waste.^1,5,16^

Despite this progress, current hydroconversion technologies rely heavily on high-pressure hydrogen (typically 20–60 bar), imposing economic, environmental, and operational constraints.^17–20^ Although emerging electrocatalytic and photocatalytic approaches offer long-term pathways toward low-carbon hydrogen, current supply remains dominated by fossil-derived sources.^21^ Moreover, hydrogen's low volumetric energy density, high flammability, and demanding storage and transport requirements complicate its deployment at scale. These limitations collectively challenge the practical sustainability of hydrogen-intensive plastic upcycling processes.^22^

Liquid organic hydrogen carriers (LOHCs) have therefore attracted increasing interest as substitutes for external supplied H_2_ in PO hydroconversion. Oxygen-containing LOHCs, such as methanol^23^ and formic acid,^24^ have been explored for *in situ* hydrogen generation, but they also introduced H_2_O- and CO_*x*_-containing reaction environments that may complicate product separation and potentially accelerate catalyst deactivation.^25^ In contrast, hydrocarbon-based LOHCs, such as decalin, provide a heteroatom-free alternative and have demonstrated the feasibility of LOHC-assisted PO conversion.^26^ Nevertheless, the solvent environment can strongly influence polymer solvation, chain conformation, hydrogenolysis kinetics, and product selectivity.^27^ Although these studies establish the general feasibility of replacing gaseous H_2_ with LOHCs, the coupled effects of LOHC molecular structure on polymer conformation, and catalytic C–C bond cleavage remain poorly understood. It also remains unclear whether LOHC-based processes can provide meaningful economic and environmental advantages over conventional H_2_-based hydroconversion at the process level.^28^

Here, we employ methylcyclohexane (MCH) as both hydrogen carrier and reaction medium for PP hydrogenolysis over bimetallic PtRu/Al_2_O_3_ (1 wt% Pt/1 wt% Ru). The PtRu catalyst couples MCH dehydrogenation with controlled C–C bond cleavage, while the MCH medium modulates PP chain mobility and access to catalytic sites. Under optimised conditions, the process affords naphtha-range hydrocarbons in yields exceeding 88 wt% at 300 °C within 5 h, outperforming most reported benchmark systems. Mechanistic analysis indicates electron coupling between Ru and Pt modulates the electronic structure of the active sites, thereby enhancing the dehydrogenation activity of Pt while preserving an optimal balance between Ru^0^ and Ru^*δ*+^ species. To elucidate the role of the hydrogen carriers, we systematically evaluate a series of hydrocarbon LOHCs and observed significant differences in catalytic performance. With the aid of molecular dynamics (MD) simulations, we attribute the variation primarily to differences in polymer–solvent interactions, which govern PP chain mobility and the accessibility of polymer segments to the catalyst surface. Complementary techno-economic analysis (TEA) and life-cycle assessment (LCA) further show that biomass-derived MCH can achieve a substantially reduced carbon intensity (0.32 kg CO_2_ per kg naphtha) and lower levelised production costs (1164 $ per ton naphtha) compared to H_2_-based process. Overall, this work provides a mechanistic and systems-level framework that integrates catalyst design, reaction engineering, polymer–solvent interactions, and sustainability assessment, thereby advancing economically and environmentally viable pathways toward a circular plastics economy.

## Results and discussions

### Tandem MCH dehydrogenation and PP hydrogenolysis

We have established an efficient tandem catalytic system using MCH as a LOHC and PP as the feedstock. PP (*M*_w_ ≈ 308 kDa) is a demanding upcycling substrate due to its lack of functional groups and also its intrinsically lower hydrogenolysis reactivity relative to PE, arising from steric constraints imposed by pendant methyl groups that hinder chain adsorption and C–C bond activation.^29^ As a baseline, a 1 wt% Pt/Al_2_O_3_ catalyst was prepared *via* an impregnation method (see ‘‘SI Methods’’), given the well-established role of Pt in alkane dehydrogenation.^3,4,30,31^ Because MCH participates directly in the tandem reaction, product yields were corrected to avoid assigning solvent-derived carbon to PP conversion. The mass of the initially charged MCH was therefore excluded from the reported product distribution by subtracting its contribution from the corresponding C_7_ fraction before calculating the final yields (see ‘‘SI Methods’’). Under standard conditions (0.2 g catalyst, 2 g PP, 0.02 mol MCH, 300 °C, stirring at 400 rpm, 5 h), Pt/Al_2_O_3_ exhibits limited activity, affording only ∼25 wt% naphtha-range products (C_5_–C_12_) while leaving a substantial fraction of unconverted polymer (∼71 wt%) (Fig. 1a and Table S1). This indicates Pt alone is insufficient to drive the tandem system. Given the tandem system requires the coupling of MCH dehydrogenation and PP hydrogenolysis, we next sought to independently enhance each elementary step. To promote hydrogen generation, Ga was introduced to form Pt3Ga/Al_2_O_3_ (1 wt% Pt/3 wt% Ga), a class of catalysts widely reported for alkane dehydrogenation.^30–32^ While Ga incorporation improves dehydrogenation activity, it does not translate into meaningful improvement in overall conversion or selectivity (Fig. S1). This observation suggests increasing hydrogen-generation capability alone is not sufficient to drive the overall tandem reaction. We therefore focused on introducing a secondary metal (*i.e.* 2 wt% Ni, Co, or Ru) to strengthen the hydrogenolysis activities. As shown in Fig. 1a, incorporation of these metals substantially improved PP conversion and liquid-hydrocarbon formation, with the overall activity following the order Pt2Ru > Pt2Co > Pt2Ni. The Pt2Ru/Al_2_O_3_ catalyst gave the highest conversion (∼91 wt%) and liquid-product yield, whereas Pt2Co/Al_2_O_3_ and Pt2Ni/Al_2_O_3_ showed more moderate activity. This trend suggests that Ru provides a better balance of C–C bond cleavage and hydrogenation–desorption steps, particularly under the present tandem conditions.^23,33,34^ In contrast, monometallic Ru/Al_2_O_3_ exhibits incomplete conversion (∼47 wt% solid residue), highlighting the need for an efficient dehydrogenation function in addition to hydrogenolysis activity.

**Fig. 1:**
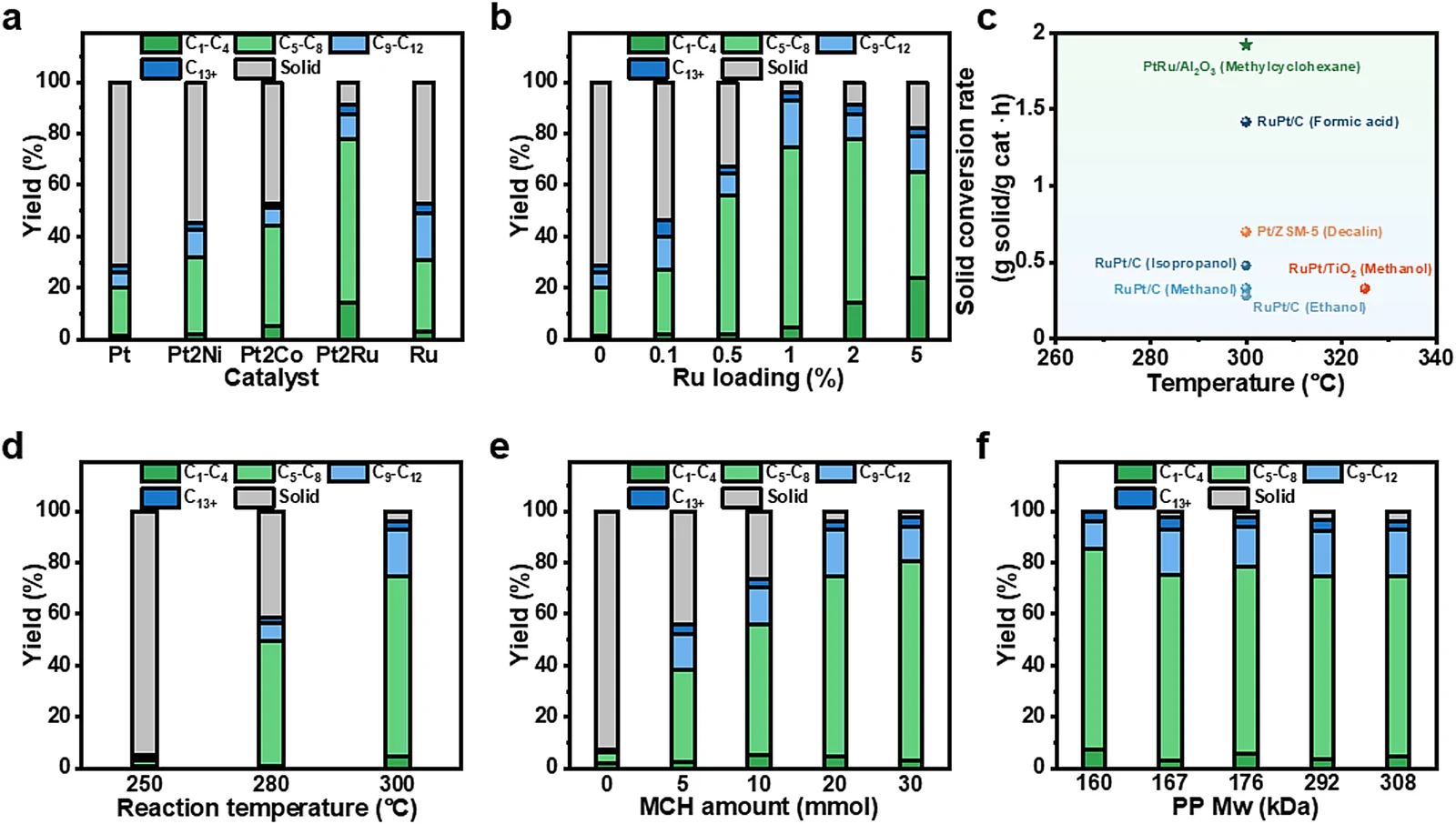
Catalytic performance of tandem MCH dehydrogenation and PP hydrogenolysis. (a) Catalytic performance of Pt, PtyM (1 wt% Pt and y wt% M) and Ru supported on γ-Al_2_O_3_. (b) Effect of Ru loading (0–5 wt%) for PtyRu/Al_2_O_3_ catalysts on reaction performance. (c) Comparison with reported LOHC-assisted hydroconversion systems from the literature.^23,24,26^ (d) Temperature dependence of catalytic performance over PtRu/Al_2_O_3_. (e) Effect of MCH amount on catalytic performance over PtRu/Al_2_O_3_. (f) Catalytic performance of PtRu/Al_2_O_3_ for PP feeds with different molecular weights. Reaction conditions: 2 g PP (*M*_w_ ∼308 kDa), 0.2 g catalyst, 300 °C, 5 h, 0.02 mol MCH, stirring at 400 rpm, 3 bar He unless otherwise specified. MCH is a reactive LOHC, the initially charged MCH was excluded from the reported product distribution. All mass balances are within the range of 97% ± 3%.

To further optimise catalyst performance, the Pt loading was initially fixed while the Ru loading was varied from 0 to 5 wt%. As shown in Fig. 1b, increasing the Ru loading from 0 to 1 wt% leads to a sharp increase in conversion, ultimately yielding >88 wt% naphtha-range products with only ∼3.8 wt% solid residue. This performance exceeds that of previously reported LOHC-assisted hydroconversion systems (Fig. 1c) and compares favourably with state-of-the-art hydrogen-based processes (Table S3). However, increasing the Ru loading to 5 wt% decreased the liquid yields and increased formation of light gases (C_1_–C_4_), while also leaving more unconverted solid. Detailed product distribution (Fig. S2) shows that excessive Ru loading broadens the product distribution and substantially increases methane formation, from below 2% over PtRu/Al_2_O_3_ to ∼16% over Pt5Ru/Al_2_O_3_. This shift indicates excess Ru disrupts the balance between MCH dehydrogenation and controlled PP hydrogenolysis, leading to over-fragmentation. The Pt loading was subsequently varied at fixed Ru loading to assess the role of the dehydrogenation function (Fig. S3). Increasing the Pt loading from 1 to 2 wt% did not noticeably improve the overall catalytic performance, consistent with the Pt3Ga results showing that enhanced dehydrogenation alone does not necessarily translate into higher PP conversion. In contrast, decreasing the Pt loading to 0.5 wt% led to a clear decline in performance, indicating that a sufficient concentration of Pt sites is required to initiate MCH dehydrogenation and sustain hydrogen availability for PP hydrogenolysis. Overall, these results demonstrate that optimal performance requires a balanced coupling between MCH dehydrogenation and PP hydrogenolysis, enabling efficient PP conversion while suppressing excessive gas formation.

The influence of reaction temperature was then examined in light of the opposing thermodynamic requirements of the tandem reactions: MCH dehydrogenation is endothermic, whereas plastic hydrogenolysis is exothermic. As shown in Fig. 1d, limited conversion is observed at 250 °C (∼95 wt% unreacted polymer), but increasing the temperature to 300 °C results in a substantial increase in conversion, consistent with the temperature dependence of MCH dehydrogenation (usually between 300 °C and 400 °C) and the requirement to provide sufficient hydrogen for PP hydroconversion.^35,36^ The role of LOHC was further investigated by varying the amount of MCH from 0 to 0.03 mol (Fig. 1e). In the absence of MCH, the reaction proceeds negligibly (>92 wt% solid residue), demonstrating the necessity of the MCH as the hydrogen source. Increasing the MCH loading from 0 to 0.02 mol leads to a pronounced increase in naphtha-range product yield and a corresponding decrease in residual polymer. Beyond this point, further increases in MCH loading do not significantly affect conversion or selectivity, indicating that hydrogen availability is no longer limiting under these conditions. Finally, the robustness of the catalytic system was evaluated using PP with varying molecular weights (as confirmed by GPC in Fig. S4 and Table S7). As shown in Fig. 1f, efficient conversion to naphtha-range hydrocarbons is achieved across all samples, with consistently low gas formation. This result demonstrates that the tandem system is insensitive to variations in polymer chain length, suggesting that hydrogen transfer and C–C cleavage kinetics remain well balanced across a broad range of substrate properties.

### Catalyst characterisation

To elucidate the geometric and electronic properties of the catalysts, a combination of structural and spectroscopic characterisation was conducted. X-ray diffraction (XRD) patterns (Fig. S5) show characteristic Bragg reflections at 2*θ* = 45.8°, 67.3°, and 84.7°, which where indexed to (400), (511), and (440) reflections of the γ-Al_2_O_3_ support.^32,37^ Bragg reflections corresponding to metallic Ru are observed at 2*θ* = 38.4°, 42.2°, 43.8°, 58.0°, 69.2°, associated with the (110), (002), (101), (102), and (110) planes, respectively.^38^ The Ru Bragg reflections have low intensity or absent at low Ru loadings but become more pronounced with increasing Ru content (*e.g.* Pt5Ru/Al_2_O_3_), indicating the progressive formation of larger or more crystalline Ru domains. In contrast, diffraction features attributable to metallic Pt are not clearly resolved, consistent with its low loading and high dispersion on the support. Inductively coupled plasma mass spectrometry (ICP-MS) analysis (Table S8) confirms that the actual metal loadings closely match the nominal values, with Pt loading ≤0.9 wt%, further supporting the absence of detectable Pt diffraction features. High-angle annular dark-field scanning transmission electron microscopy (HAADF-STEM) images (Fig. 2a and Fig. S6) reveal a highly dispersed metal phase, with PtRu nanoparticles of approximately 1.0 nm in diameter. Energy-dispersive X-ray spectroscopy (EDS) elemental mapping (Fig. 2b) shows that Pt and Ru are co-localised within these nanoparticles, indicating the formation of bimetallic PtRu domains rather than segregated monometallic phases.

**Fig. 2:**
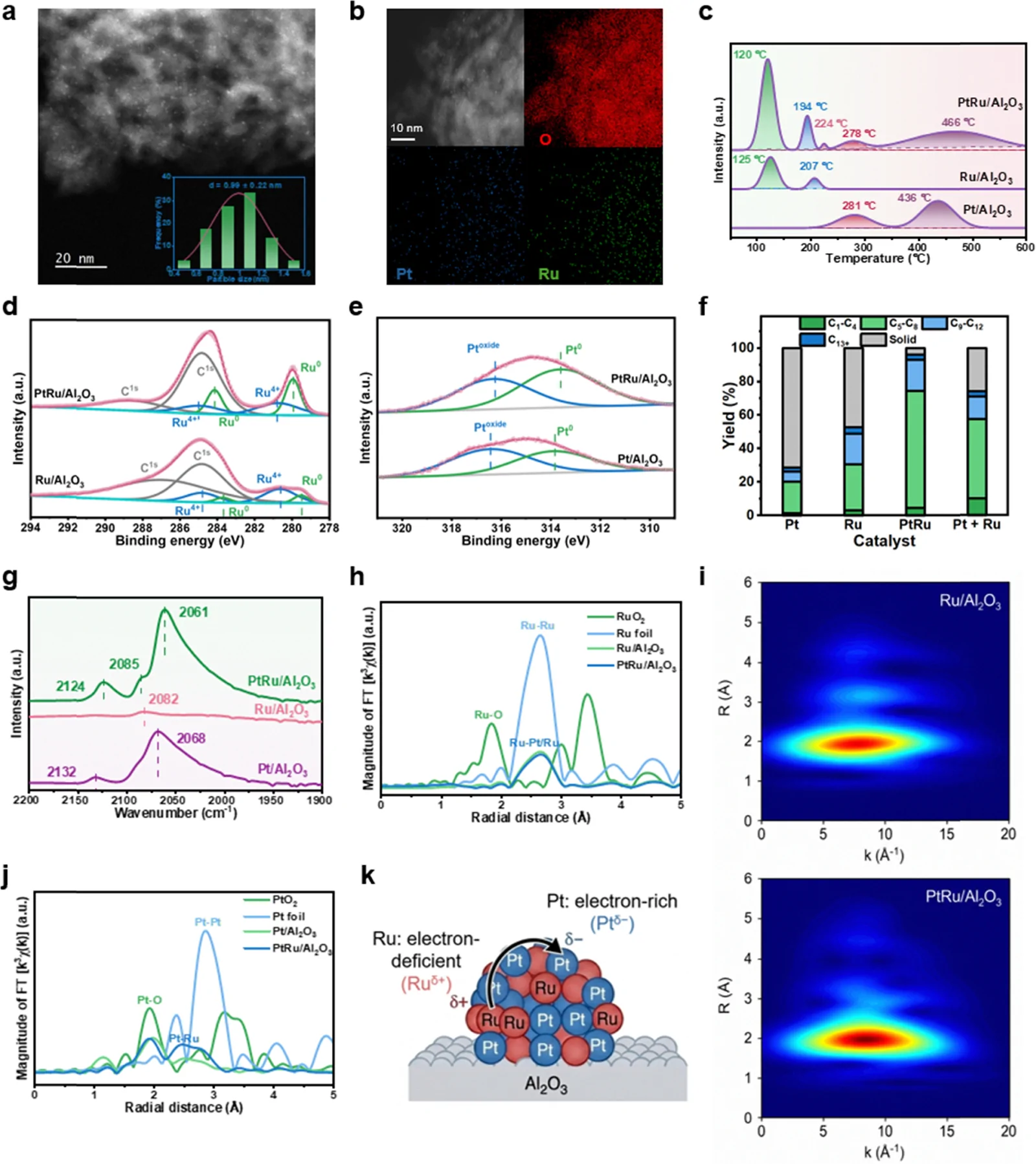
Structural and electronic characterisation of the catalysts. (a) HAADF-STEM image of PtRu/Al_2_O_3_ catalyst with corresponding particle size distribution. (b) EDS elemental mapping of PtRu/Al_2_O_3_ (red: O, blue: Pt, and green: Ru). (c) H_2_-TPR profiles of PtRu/Al_2_O_3_ compared with Pt/Al_2_O_3_ and Ru/Al_2_O_3_. (d) Ru 3d XPS spectra of PtRu/Al_2_O_3_ and Ru/Al_2_O_3_. (e) Pt 4d XPS spectra of PtRu/Al_2_O_3_ and Pt/Al_2_O_3_. (f) Catalytic performance of physical mixture of monometallic catalysts and bimetallic catalysts. (g) CO adsorption FTIR of PtRu/Al_2_O_3_, Pt/Al_2_O_3_, and Ru/Al_2_O_3_. (h) and (i) Fourier-transform EXAFS spectra of Ru K-edge and corresponding wavelet-transform EXAFS maps for Ru/Al_2_O_3_ and PtRu/Al_2_O_3_. (j) Fourier-transform EXAFS spectra of Pt L_3_-edge. (k) Schematic illustration of electron interaction in bimetallic PtRu domains.

To further probe metal–metal interactions, H_2_ temperature-programmed reduction (H_2_-TPR) was performed on PtRu/Al_2_O_3_ and the corresponding monometallic catalysts. As shown in Fig. 2c, Pt/Al_2_O_3_ exhibits two reduction peaks at 281 and 436 °C, assigned to the reduction of surface PtO_*x*_ species weakly interacting with the support and highly dispersed PtO_*x*_ species strongly interacting with the support.^39^ In contrast, Ru/Al_2_O_3_ displays reduction peaks at 125 and 207 °C, corresponding to well-dispersed Ru species and bulk RuO_2_, respectively.^40^ Upon formation of the bimetallic PtRu/Al_2_O_3_ catalyst, the H_2_-TPR profile changes markedly. Relative to Ru/Al_2_O_3_, the reduction peaks shift to lower temperatures and exhibit substantially increased intensities, indicating enhanced reducibility and improved dispersion of Ru species in the presence of Pt. The pronounced increase in the low-temperature reduction peak suggests that Pt promotes the formation of highly dispersed Ru species while suppressing the formation of bulk RuO_2_. Concomitantly, the reduction behavior of Pt is also modified. The relative intensity of the high-temperature reduction peak increases at the expense of the low-temperature peak, indicating a higher fraction of PtO_*x*_ species strongly interact with the support. This redistribution implies that Ru promotes the stabilisation of highly dispersed Pt species. In addition, a new reduction peak appears at ∼224 °C in the bimetallic catalyst, located between the characteristic reduction temperatures of Pt and Ru species. This intermediate peak is probably attributed to the reduction of Pt–Ru interfacial species, providing further evidence for the formation of bimetallic PtRu domains.^24^

X-ray photoelectron spectroscopy (XPS) of the Ru 3d XPS and Pt 4d regions (Fig. 2d and e) provides further evidence for electronic interaction between Pt and Ru in the bimetallic PtRu/Al_2_O_3_ catalyst. Compared to the monometallic counterparts, the Ru 3d peaks shift to higher binding energy by ∼0.4 eV, while the Pt 4d peaks shift to lower binding energy by ∼0.3 eV. These complementary shifts are consistent with electron redistribution between the two metals, in agreement with previous reports,^41^ resulting in electron-enriched Pt sites and electron-deficient Ru species. In the context of the tandem reaction, such electronic modification is expected to facilitate MCH dehydrogenation on Pt sites, while moderating excessive C–C bond cleavage during PP hydrogenolysis over Ru sites.^18,32,42^ The physical mixture of Pt/Al_2_O_3_ and Ru/Al_2_O_3_ was further examined to distinguish the effect of bimetallic proximity from the simple coexistence of Pt and Ru (Fig. 2f). The physical mixture exhibits substantial conversion, confirming that spatially separated Pt and Ru sites can already promote the tandem sequence to a significant extent. Although the physical mixture remains active, it gives higher gas formation (10.1 wt%) and a larger fraction of residual solid (25.6 wt%) than PtRu/Al_2_O_3_ (4.5 wt% gas and 3.8 wt% solid). This comparison indicates that close Pt–Ru proximity improves the balance between MCH dehydrogenation and PP hydrogenolysis, although the physical-mixture test alone does not establish the electronic origin of this effect.

CO adsorption Fourier-transform infrared spectroscopy (CO-FTIR) further corroborates these electronic effects under *in situ* conditions (Fig. 2g). After *in situ* reduction in 5 vol% H_2_/N_2_ at 400 °C for 3 h, cooling to room temperature, exposure to CO for 1 h, and evacuation for 1 h to remove physiosorbed species, monometallic Pt/Al_2_O_3_ exhibits bands at 2068 and 2132 cm^−1^, corresponding to linearly adsorbed CO on metallic Pt^0^ and oxidized Pt species, respectively,^43^ whereas Ru/Al_2_O_3_ shows a single band at 2082 cm^−1^ associated with CO adsorption on highly dispersed Ru sites.^44^ In the bimetallic catalyst, the Pt-related bands shift to lower wavenumbers (2061 and 2124 cm^−1^), indicating increased electron density at Pt sites, while the Ru band shifts slightly to a higher wavenumber (2085 cm^−1^), consistent with electron depletion at Ru. These shifts provide direct *in situ* spectroscopic evidence of electronic coupling between Pt and Ru.

The atomic-scale structure of the bimetallic catalyst was further elucidated by extended X-ray adsorption fine structure (EXAFS) spectroscopy. In the *k*^3^-weighted Fourier-transform Ru K-edge spectra (Fig. 2h), peaks at ∼1.8 Å and ∼2.5 Å correspond to Ru–O and Ru–Ru scattering, respectively, consistent with RuO_2_ and Ru foil references.^45^ Both Ru/Al_2_O_3_ and PtRu/Al_2_O_3_ exhibit a dominant peak at ∼2.5 Å, reflecting overlapping contributions from Ru–Pt and Ru–Ru scattering.^24^ To resolve these contributions, wavelet-transform EXAFS maps (Fig. 2i) were examined. PtRu/Al_2_O_3_ shows a clear change in the scattering distribution relative to Ru/Al_2_O_3_, consistent with a modified Ru coordination environment and the presence of heavier neighboring atoms. This conclusion was further corroborated by Pt L_3_-edge analysis (Fig. 2j). In addition to Pt–O (∼1.9 Å) and Pt–Pt (∼2.8 Å) scattering contributions, the bimetallic catalyst exhibits an additional peak at ∼2.5 Å that is absent in Pt/Al_2_O_3_, which is assigned to Pt–Ru coordination in agreement with previous studies.^46,47^ Collectively, these results indicate that PtRu forms electronically coupled bimetallic domains (Fig. 2k), which modify the local electronic structure of the active sites and contribute to the improved performance observed in tandem MCH dehydrogenation and PP hydrogenolysis.

### Mechanistic coupling, stability, and scalability of the tandem system

To elucidate the coupling between MCH dehydrogenation and PP hydrogenolysis, we quantified the hydrogen released *in situ* from MCH and examined it in the context of the thermodynamic behaviour of the tandem system. Direct measurement of H_2_ in the presence of PP is challenging because a substantial fraction of the hydrogen generated from MCH is consumed during PP hydrogenolysis. Nevertheless, MCH dehydrogenation over PtRu/Al_2_O_3_ proceeds with high selectivity to toluene (94.6%) in the absence of PP under identical conditions (Fig. S7), and control experiments using external H_2_ showed no detectable formation of MCH during PP hydrogenolysis. Hydrogen release from MCH can therefore be estimated reliably from MCH conversion and toluene yield. As shown in Fig. 3a, the blank experiment gave 0.02 mol MCH conversion of 9.9%, corresponding to 5.87 mmol H_2_ from the stoichiometry of MCH dehydrogenation; GC analysis of the reactor after reaction gave 5.72 mmol H_2_, in close agreement with this estimate. This low conversion reflects the thermodynamic limitations of MCH dehydrogenation: the reaction is endothermic and typically requires temperature above 350 °C to reach substantial conversion.^35,36^ Additionally, operation in a closed, constant-volume reactor raises the system pressure upon heating and disfavours the forward reaction due to the increase in the number of gas-phase molecules, as predicted by Le Chatelier's principle. Theoretical calculations and literature data further indicate an equilibrium conversion of approximately 11% under these conditions (Fig. 3a and Fig. S8).^48^ In contrast, when PP was introduced, MCH conversion increased to 29.5%, corresponding to 17.0 mmol H_2_ released from MCH. Based on the detailed product distribution (Fig. S2) and the degree of unsaturation of the PP-derived products, the hydrogen consumed for PP hydrogenolysis was estimated to be 15.8 mmol, leaving a net hydrogen surplus of 1.21 mmol, which matches well with the 1.15 mmol H_2_ measured by GC after reaction. This increase in MCH conversion arises from continuous hydrogen consumption during PP hydrogenolysis, which lowers the hydrogen partial pressure and shifts the dehydrogenation equilibrium toward product formation. Overall, these results show that hydrogen generation from MCH and hydrogen consumption during PP hydrogenolysis are tightly coupled. Accordingly, the tandem process operates beyond the nominal equilibrium limit for MCH dehydrogenation under closed conditions.

**Fig. 3:**
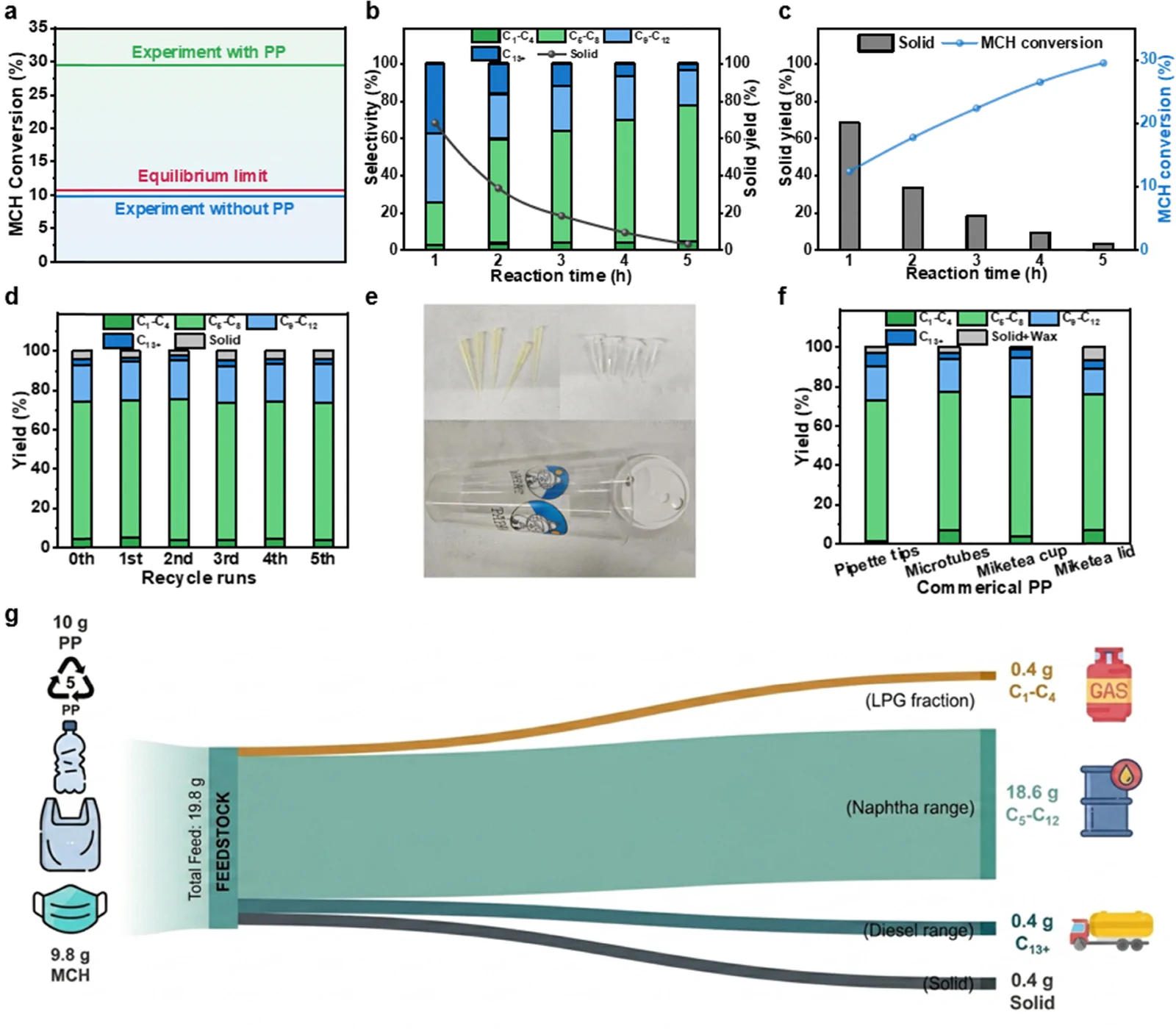
Mechanistic coupling, stability, and scalability of the tandem system. (a) MCH conversion under different reaction conditions. (b) Time-dependent product distribution over PtRu/Al_2_O_3_ catalyst. (c) Time-dependent changes in MCH conversion and solid yield. (d) Catalyst stability over multiple reaction cycles. (e) Representative commercial plastic samples, including pipette tips, microtubes, and miketea packaging. (f) Catalytic performance of PtRu/Al_2_O_3_ for real plastic feedstocks. (g) Mass-flow distribution for the scaled-up conversion of 10 g PP with 9.8 g MCH.

Time-dependent experiments over PtRu/Al_2_O_3_ (Fig. 3b and Table S9) provide further insight into reaction dynamics. As the reaction progresses, the solid residue decreases steadily, accompanied by a shift in product distribution from heavier hydrocarbons (C_13+_) towards naphtha-range products (C_5_–C_12_), consistent with sequential PP hydrogenolysis and secondary cracking of PP-derived intermediates. The hydrogen-transfer dynamics were further examined by monitoring the autoclave pressure during reaction (Fig. S9). Upon heating to 300 °C, the system pressure reaches ∼35 bar, and decreases to ∼32 bar within the first hour before stabilizing at ∼31 bar for the remaining reaction period. Notably, this relatively constant pressure contrasts with the continuous consumption of polymer (Fig. 3b). This relatively stable pressure profile indicates that hydrogen consumed during PP hydrogenolysis is replenished by *in situ* hydrogen generation from MCH dehydrogenation. The hydrogen-balance calculation further supports this interpretation, showing that most of the hydrogen released from MCH is consumed during PP hydrogenolysis, with only a small residual amount remaining after reaction. Overall, these results indicate that the tandem process operates under a quasi-steady-state hydrogen regime, in which hydrogen generation and consumption remain closely coupled within the closed reactor. This mechanism differs fundamentally from formic acid-assisted systems, where rapid decomposition of the hydrogen donor generates a transient high-pressure hydrogen environment at the initial stage.^24^ In contrast, the gradual increase in MCH conversion with reaction time (Fig. 3c) further confirms continuous hydrogen release and utilisation throughout the reaction. Despite this, the overall hydrogen partial pressure remains lower than that typically required for conventional hydrogen-driven hydrogenolysis (≥20 bar H_2_ at room temperature).^4,14^ This observation suggests that hydrogen is likely utilised efficiently at the catalyst surface, probably through rapid adsorption and transfer within the bimetallic PtRu active sites, thereby establishing a locally hydrogen-rich environment that enables efficient C–C bond cleavage without the need for high bulk hydrogen pressures.

Catalyst stability was evaluated over multiple reaction cycles (Fig. 3d). PtRu/Al_2_O_3_ maintained comparable PP conversion and product selectivity over six consecutive runs, indicating good stability under the reaction conditions. Post-reaction characterization of the spent catalyst further supports its structural stability (Fig. S10 and S11). HAADF-STEM images of the fresh and spent catalysts, together with the corresponding particle-size distributions, show no obvious nanoparticle sintering after cycling. TGA indicates only a small amount of carbonaceous residue, with coke formation remaining at about 1.1 wt% after the first run and 1.2 wt% after six cycles, suggesting negligible coke accumulation under the present conditions. ICP analysis also shows minimal metal loss after recycling: the Pt and Ru contents change only slightly from 0.89 and 0.85 wt% in the fresh catalyst to 0.86 and 0.82 wt% after cycling, respectively, corresponding to an essentially unchanged Pt/Ru ratio. Overall, these results indicate that the bimetallic catalyst largely retains its structural integrity during repeated tandem MCH dehydrogenation–PP hydrogenolysis cycles.

The applicability of the catalyst to realistic feedstocks was assessed using a range of commercial plastic materials (Fig. 3e), including laboratory consumables (pipette tips and microtubes) and consumer products (*e.g.*, tea cups and lids). As shown in Fig. 3f, these materials exhibit conversion and selectivity comparable to those obtained with a virgin PP feedstock (Fig. 1), demonstrating the robustness of the catalytic system towards practical plastic waste streams. The process was subsequently examined at a fivefold larger batch scale, limited by the 50 mL autoclave volume. Under these conditions (10 g PP; 0.1 mol MCH (∼9.8 g); and 1 g PtRu/Al_2_O_3_ catalyst), with all other reaction parameters constant, the product distribution remained similar to that observed at smaller scale (Fig. S12) on the PP-carbon basis. As shown in Fig. 3g, the reaction produced approximately 0.4 g of C_1_–C_4_ gases (LPG fraction), 18.6 g of C_5_–C_12_ liquid hydrocarbons, and 0.4 g of C_13+_ products, with carbon from PP and MCH accounted for together, highlighting the potential of the tandem system for scalable plastic upcycling.

### Solvent effects in the tandem system

Although LOHCs have been widely explored to assist PO hydrogenolysis,^23,24,26^ the broader role of the liquid medium in tandem dehydrogenation–hydroconversion systems remains poorly understood. Beyond supplying hydrogen, the LOHCs may affect polymer swelling, chain dynamics, hydrogen availability, and mass transport. We therefore examined whether the enhanced performance of the MCH-assisted system could be attributed solely to its hydrogen-donor capacity. As an initial comparison, we replaced MCH with an equivalent amount of external hydrogen corresponding to its hydrogen capacity (0.02 mol MCH ≈ 29 bar H_2_ at room temperature) and performed direct hydrogenolysis under otherwise identical conditions. As shown in Fig. 4a, the hydrogen-fed system exhibits significantly lower activity, yielding ∼31 wt% solid compared to only 3.8 wt% under MCH conditions. Achieving a comparable conversion required either extending the reaction time to 10 h or increasing the hydrogen pressure to 45 bar. These results indicate that MCH does not act only as a hydrogen donor, but also enhances reaction kinetics, likely through improved polymer solvation and transport.

**Fig. 4:**
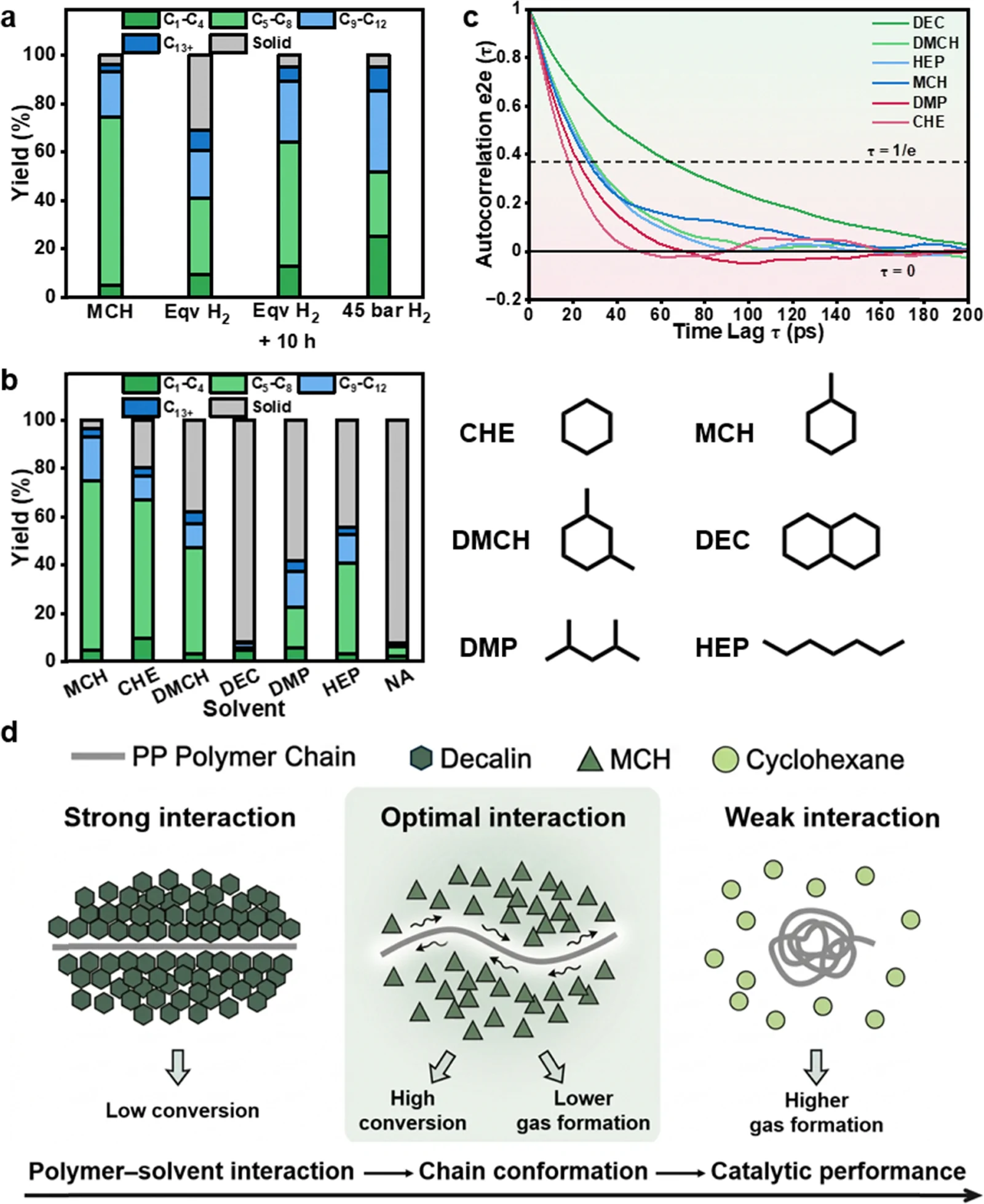
Solvent effects in the tandem system. (a) Catalytic performance under MCH-assisted conditions compared with external hydrogen supply. (b) Catalytic performances in the presence of different solvents (0.02 mol) and their corresponding molecular structures; NA denotes reactions conducted without added solvent. (c) End-to-end autocorrection functions of PP chains in different solvents. (d) Schematic illustration of solvent-polymer interaction and their influence on catalytic performance.

To further investigate the influence of the liquid medium, a series of hydrocarbon-LOHCs and model solvents were evaluated. Surprisingly, substantial differences in catalytic performance were observed using different LOHCs. As shown in Fig. 4b, MCH achieved near-complete conversion, whereas cyclohexane and 1,3-dimethylcyclohexane gave conversions of 80% and 68%, respectively. In contrast, decalin resulted in less than 8% PP conversion. Owing to these LOHCs exhibiting distinct dehydrogenation activities and generating different hydrogen partial pressures, their hydrogen-release behavior was examined independently under the reaction conditions. As shown in Fig. S13, cyclohexane exhibited the highest conversion of 11.1%, followed by MCH (9.9%) and 1,3-dimethylcyclohexane (8.6%), consistent with the corresponding hydrogen yields. Decalin showed the lowest dehydrogenation conversion, but the differences in hydrogen generation among these carriers were modest compared with the much larger differences in PP conversion. In particular, the contrast between near-complete PP conversion with MCH and the less than 8% conversion with decalin cannot be explained solely by hydrogen yield. These control experiments therefore indicate that hydrogen-release capacity is not the only factor governing the LOHC-dependent catalytic performance.

The differences in residual polymer were further supported by GPC analysis of residual solids (Fig. S14 and Table S13), which shows that *M*_w_ of residues decreases to <3 kDa for MCH, but remains >44 kDa for decalin, indicating much more limited polymer decomposition over decalin. To further probe structure-dependent liquid-medium effects without differences in chemical composition, the structural isomers *n*-heptane and 2,4-dimethylpentane were also examined. Despite their identical molecular formula, these two solvents produced distinct PP conversions and product distributions, providing additional evidence that solvent molecular structure affects the tandem reaction.

To gain further insight into solvent–polymer interactions, non-reactive MD simulations were performed to evaluate the behavior of PP chains in different solvents under reaction-relevant conditions (Fig. 4c and Table 1; see section 1.5 of the SI for more details on the simulations). These simulations do not explicitly include the polymer adsorption, C–C bond cleavage, solvent dehydrogenation, or hydrogen transfer; they are therefore used here to provide qualitative descriptors of solvent-dependent polymer conformation and mobility rather than direct mechanistic proof of catalytic reactivity. Among the solvents examined, decalin exhibits markedly slower end-to-end autocorrelation dynamics for the polymer compared to the other solvents, indicating hindered chain mobility. It also gave the largest radius of gyration (*R*_g_) and the lowest root mean square deviation (RMSD), consistent with strong polymer–solvent interactions that stabilise extended, relatively rigid polymer conformations. Such “sticky” polymer–solvent interactions may hinder polymer diffusion and limit access to catalytic sites, providing a plausible explanation for the low conversion observed in decalin.

**Table 1:** Summary of MD simulation results over different solvents

Solvent	Relaxation time (ps)	*R*_g_ (Å)	RMSD (Å)
DEC	64.69	10.87	7.68
DMCH	29.64	10.53	8.04
HEP	28.48	10.4	8.22
MCH	27.15	10.35	9.06
DMP	22.56	10.12	7.94
CHX	18.24	10.01	8.3

Across the solvent series, MD-derived results showed qualitative correlations with the experimental product distributions. Shorter relaxation times and smaller *R*_g_ values, which indicate more compact and coiled polymer conformations, were associated with higher formation of light gaseous products. For example, although MCH, cyclohexane, and 1,3-dimethylcyclohexane provide comparable hydrogen-carrier capacities, cyclohexane gave the highest C_1_–C_4_ gas fraction, followed by MCH and then 1,3-dimethylcyclohexane. This trend broadly follows their relaxation time and *R*_g_ ordering. A similar relationship was observed for the structural isomers: 2,4-dimethylpentane showed a shorter relaxation time and smaller *R*_g_ than *n*-heptane, consistent with a more compact chain conformation and greater gas formation relative to the linear isomer.

RMSD values, which quantify the average structural deviation of polymer chains from a reference configuration, provide a complementary descriptor of conformational flexibility and correlate with overall polymer conversion. Compared with cyclohexane and 1,3-dimethylcyclohexane, MCH exhibited the highest RMSD values, indicating greater conformational flexibility of the polymer chains. This flexibility may facilitate polymer transport and adsorption at catalytic sites, thereby promoting higher conversion. A similar trend was observed for *n*-heptane and 2,4-dimethylpentane: the linear solvent, which gave a higher RMSD value, also afforded higher PP conversion than the branched isomer. Together, the experimental and simulation results indicate that solvent selection plays an important role in tandem PP hydroconversion. The solvent affects not only hydrogen availability, but also polymer swelling, chain mobility, and product selectivity. Under the present reaction conditions, MCH appears to provide a favorable balance between hydrogen-donor capability, polymer-chain flexibility, and moderate chain extension. This balance enables efficient PP conversion while limiting excessive fragmentation to low-value C_1_–C_4_ gases. These findings highlight the importance of considering the liquid medium as an active design parameter in LOHC-assisted plastic upcycling, rather than treating it only as a hydrogen source.

### Techno-economic analysis and life-cycle assessment

To evaluate the economic and environmental feasibility of the tandem MCH dehydrogenation and PP hydrogenolysis process, the experimental data were scaled to a process-level model (Fig. S15), and comprehensive TEA and LCA were performed following established system-level assessment frameworks.^49,50^ The MCH-assisted process was benchmarked against a conventional hydrogen-based route for naphtha production. As shown in Fig. 5a, the cradle-to-gate carbon footprint depends strongly on MCH origin. For the MCH-assisted process, it decreases from 2.05 kg CO_2_e per kg naphtha with fossil-derived MCH to 0.32 kg CO_2_e per kg naphtha in the biomass-derived proxy scenario. These endpoints correspond to the assumed MCH carbon-intensity range of 0.59–3.73 kg CO_2_e per kg MCH. In contrast, the hydrogen-based route shows only a modest reduction when switching from grey to green hydrogen (0.79 to 0.55 kg CO_2_ per kg naphtha), as emissions are dominated by direct process contributions rather than hydrogen production. This is attributed to limited carbon selectivity, where a significant fraction of carbon is converted to light hydrocarbons (C_1_–C_4_) and subsequently combusted. These findings highlight that the MCH pathway is feedstock-limited, whereas the hydrogen-based route is process-limited. Moreover, feedstock-related emissions can be mitigated through feedstock substitution and geographic sourcing, whereas process-limited emissions arise from intrinsic selectivity constraints, making them more challenging to reduce through system-level adjustments.

**Fig. 5:**
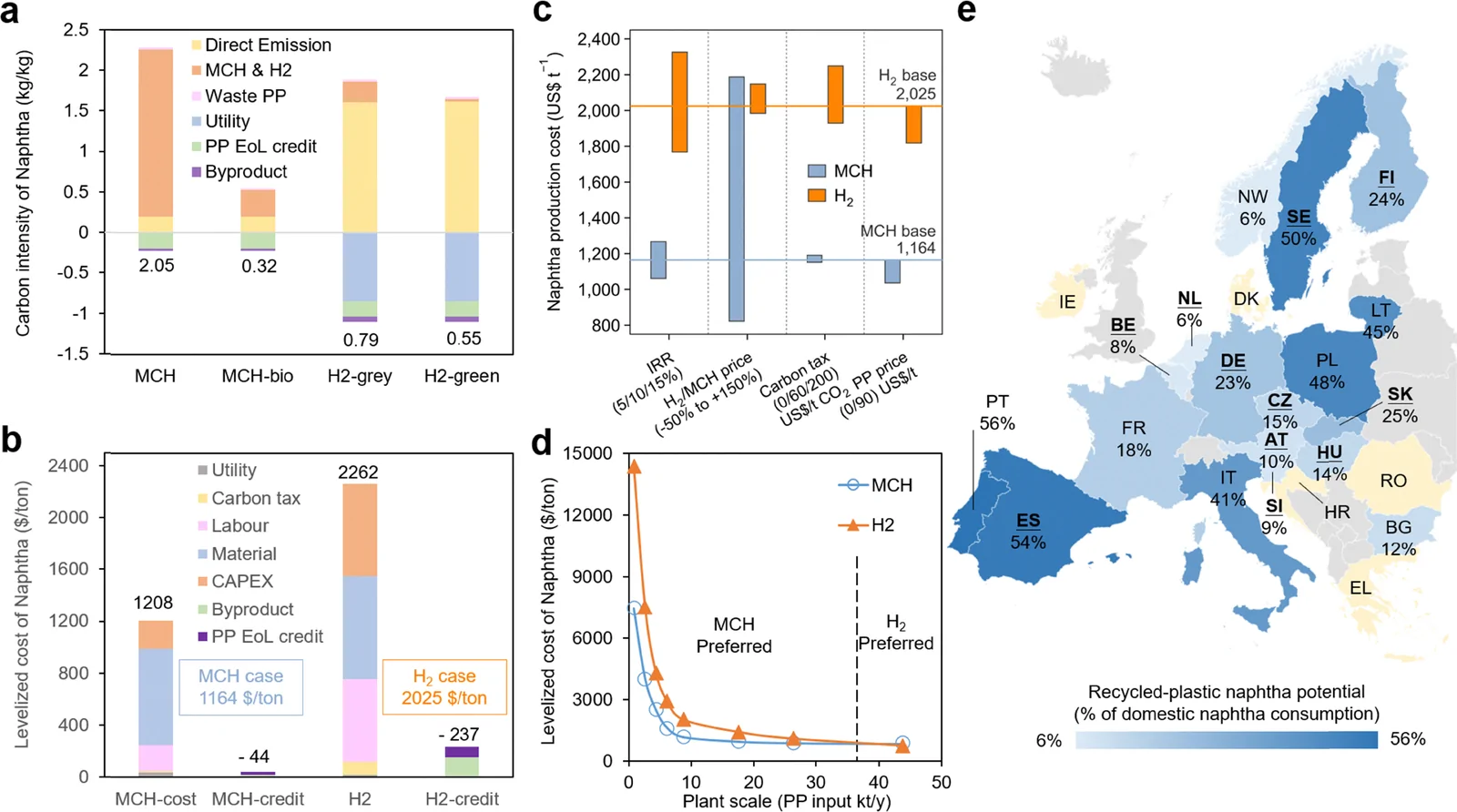
Techno-economic and environmental assessment of MCH-based and H_2_-based naphtha production. (a) Cradle-to-gate carbon footprint under different feedstock sourcing scenarios; PP downstream credit represents avoided emissions from weighted end-of-life treatment under current conditions. (b) Levelized cost of naphtha production using conventional feedstocks; landfill deduction represents avoided disposal costs when collected PP is diverted from landfill. (c) Sensitivity analysis of the levelised cost of naphtha production. (d) Sensitivity analysis of naphtha production costs to plant capacity. (e) Country-level assessment of potential naphtha production from recycled plastics, expressed as a percentage of total naphtha consumption in Europe. Note: (1) bold and underlined countries are import-dependent due to insufficient domestic naphtha production. (2) Yellow-highlighted countries indicate recycled-plastic-derived naphtha capacity exceeding current domestic production or consumption. (3) Unreacted MCH present in the product stream is excluded from net naphtha production. (4) Baseline assumes all recycled plastics; PP-only scenario scaled by 0.185 (PP fraction in total plastic waste).

The economic performance of the two processes was further compared (Fig. 5b). The MCH-based process exhibits a lower levelised cost of naphtha production ($1164 per ton) due to the higher naphtha selectivity and lower operating pressure compared to the H_2_-based process ($2025 per ton). Sensitivity analysis (Fig. 5c and d) indicates that the MCH-based process is less sensitive to variations in key economic parameters such as cost of PP, IRR, and plant size. Owing to its higher naphtha yield, these inputs are effectively diluted over a larger product output, leading to a lower unit production cost and reduced sensitivity to such parameters.^51^ Among the sensitivity cases examined, only MCH price can reverse the cost advantage of the MCH-assisted route, highlighting the importance of MCH pricing, particularly given the uncertainty surrounding bio-based MCH costs. The MCH-based process is economically favorable at smaller scales (<∼40 kt year^−1^ PP throughput), whereas the H_2_-based process becomes more competitive at larger scales. From a practical perspective, the centralised accumulation of large volumes of plastic waste is logistically challenging, particularly given the distributed nature of plastic waste generation and waste valorization systems.^52^ This suggests that decentralised or modular processing units based on the MCH-assisted route may offer a more viable strategy for real-world implementation than large-scale, H_2_-intensive facilities. Finally, a country-level assessment was conducted to evaluate the potential contribution of recycled plastic-derived naphtha to domestic demand across Europe (Fig. 5e). The results reveal substantial geographic heterogeneity and distinct system roles across countries. Import-dependent countries with limited domestic naphtha production (*e.g.*, Spain and Sweden) could significantly benefit from localised plastic-to-naphtha conversion, improving supply security and reducing reliance on imports. In contrast, self-sufficient countries may adopt plastic upcycling primarily based on economic and policy drivers rather than supply constraints. Notably, in some countries (*e.g.*, Denmark and Ireland), the potential production of plastic-derived naphtha exceeds current domestic demand, indicating opportunities for export and the development of regional circular feedstock supply networks. These findings reveal a structural mismatch between plastic waste availability and petrochemical demand across Europe, highlighting the need for cross-border integration to fully realise the benefits of plastic upcycling.^53^ Overall, the combined TEA, LCA, and country-level analyses identify MCH-assisted tandem conversion as a process-intensified strategy that enhances economic robustness through yield-driven cost dilution and achieves low carbon intensity when integrated with renewable feedstocks, while enabling regional integration of plastic-derived naphtha supply to address imbalances between waste availability and petrochemical demand.

## Conclusions

In summary, we report a tandem catalytic process for upcycling PP (*M*_w_ ≈ 308 kDa); the process elegantly couples MCH dehydrogenation with PP hydrogenolysis, enabling efficient upcycling without the use of added hydrogen. The bimetallic PtRu/Al_2_O_3_ catalyst exhibits pronounced electronic synergy, where generates electron-enriched Pt sites and electron-deficient Ru species. This electronic modulation enhances MCH dehydrogenation to supply hydrogen *in-situ*, while moderates excessive C–C bond cleavage for gas formation during PP hydrogenolysis. Importantly, the role of the MCH solvent extends beyond acting as a hydrogen supplier: MD analysis reveals that polymer–solvent interactions govern chain conformation and accessibility to catalytic sites, thereby controlling activity and product selectivity. TEA and LCA further show that MCH-assisted process, particularly using renewable feedstocks, can achieve lower carbon intensity and competitive costs relative to H_2_-based processes. These findings establish MCH-assisted tandem catalysis as a viable strategy for scalable and sustainable plastic upcycling, advancing both the waste management and circular economy.

## Author contributions

D. O. H. and H. W. conceived the project. D. O. H. secured the funding. H. W. performed the experimental work with contributions from F. Z. T. Y., J. L., Y. L. and R. C. T.-R. Z. Z. conducted the TEA and LCA with support from J. P. M. L. carried out the MD simulations with input from Z. W., R. L. and Y. L. D. O. H. supervised the experimental work, A. Y. supervised the TEA and LCA, and M. S. supervised the MD simulations. H. W., Z. Z. and M. L. wrote the manuscript with input from all authors. All authors discussed the results, commented on the manuscript, and approved the final version.

## Conflicts of interest

There are no conflicts to declare.

## Supplementary Material

EE-OLF-D6EE05130H-s001

## Data Availability

The supporting data have been provided as part of the supplementary information (SI). Supplementary information is available. See DOI: https://doi.org/10.1039/d6ee05130h.

## References

[cit1] Geyer R., Jambeck J. R., Law K. L. (2017). Sci. Adv..

[cit2] MacLeod M., Arp H. P. H., Tekman M. B., Jahnke A. (2021). Science.

[cit3] Zheng K., Wu Y., Hu Z., Wang S., Jiao X., Zhu J., Sun Y., Xie Y. (2023). Chem. Soc. Rev..

[cit4] Sun J., Dong J., Gao L., Zhao Y.-Q., Moon H., Scott S. L. (2024). Chem. Rev..

[cit5] *Plastics – the fast Facts 2025*, Plastics Europe, Statista, 2025

[cit6] Korley L. T. J., Epps T. H., Helms B. A., Ryan A. J. (2021). Science.

[cit7] Stegmann P., Daioglou V., Londo M., van Vuuren D. P., Junginger M. (2022). Nature.

[cit8] Ellis L. D., Rorrer N. A., Sullivan K. P., Otto M., McGeehan J. E., Román-Leshkov Y., Wierckx N., Beckham G. T. (2021). Nat. Catal..

[cit9] Jehanno C., Alty J. W., Roosen M., De Meester S., Dove A. P., Chen E. Y. X., Leibfarth F. A., Sardon H. (2022). Nature.

[cit10] Chu M., Tu W., Zhang Q., Huang W., Chen J., Ma D. (2025). Nat. Rev. Clean Technol..

[cit11] Wang H., Edman Tsang S. C. (2024). Cell Rep. Phys. Sci..

[cit12] Wang H., Huang S., Tsang S. C. E. (2025). Chem. Commun..

[cit13] Yuan F., Ran H., Chen T., Jing Y. (2025). ACS Catal..

[cit14] Wang H., Yang F. Z. T., Juntarawatt S., Li Y., Turnell-Ritson R. C. (2026). Appl. Catal., A.

[cit15] Ran H., Shen R.-J., Ni W., Zheng M., Sun X., Zhang S., Wang Z.-Q., Zheng S., Qu X., Jing Y. (2026). Energy Environ. Sci..

[cit16] Celik G., Kennedy R. M., Hackler R. A., Ferrandon M., Tennakoon A., Patnaik S., LaPointe A. M., Ammal S. C., Heyden A., Perras F. A., Pruski M., Scott S. L., Poeppelmeier K. R., Sadow A. D., Delferro M. (2019). ACS Cent. Sci..

[cit17] Nakaji Y., Tamura M., Miyaoka S., Kumagai S., Tanji M., Nakagawa Y., Yoshioka T., Tomishige K. (2021). Appl. Catal., B.

[cit18] Wang H., Yoskamtorn T., Zheng J., Ho P.-L., Ng B., Tsang S. C. E. (2023). ACS Catal..

[cit19] Wang H., Hare D. O. (2026). Discover Chem..

[cit20] Wang H., Li Y., Tsang S. C. E. (2025). ACS Sustain. Chem. Eng..

[cit21] Global Hydrogen Review 2025, IEA, Paris, 2025

[cit22] Faye O., Szpunar J., Eduok U. (2022). Int. J. Hydrogen Energy.

[cit23] Lee J., Kwon T., Hyuk Kang K., Won W., Ro I. (2025). Angew. Chem., Int. Ed..

[cit24] Wang Y., Hu Q., Qian S., Zhao J., Cheng Y., Ma J., Zhang J., Niu Z. (2025). Nat. Commun..

[cit25] Sun L., Wang X., Xu J., Zhao M., Song S., Zhang H., Wang X. (2026). J. Am. Chem. Soc..

[cit26] Kwon T., Ahn S., Kim T., Kang J. H., Jeong S., Kang K. H., Won W., Ro I. (2025). Nat. Commun..

[cit27] Jia C., Xie S., Zhang W., Intan N. N., Sampath J., Pfaendtner J., Lin H. (2021). Chem. Catal..

[cit28] Yadav G., Uekert T., DesVeaux J. S., Van Roijen E. C., Rorrer J. E., Brenner A. E., Drake G., Zichittella G., Carlson N., Román-Leshkov Y., Beckham G. T. (2026). Joule.

[cit29] Tu W., Chu M., Yan T., Zhang Q., Wang X., Zhang L., Cao M., Zhang Q., Zhu M., Zhan M., Chen J. (2026). Nat. Commun..

[cit30] Chen S., Chang X., Sun G., Zhang T., Xu Y., Wang Y., Pei C., Gong J. (2021). Chem. Soc. Rev..

[cit31] Carter J. H., Bere T., Pitchers J. R., Hewes D. G., Vandegehuchte B. D., Kiely C. J., Taylor S. H., Hutchings G. J. (2021). Green Chem..

[cit32] Wang H., Pan X., Wang Y., Ng B. K. Y., Tsang S. C. E. (2025). Appl. Catal., A.

[cit33] Almithn A. S., Hibbitts D. D. (2020). ACS Catal..

[cit34] Hu Q., Qian S., Wang Y., Zhao J., Jiang M., Sun M., Huang H., Gan T., Ma J., Zhang J., Cheng Y., Niu Z. (2024). Nat. Commun..

[cit35] He Z., Li K., Chen T., Feng Y., Villalobos-Portillo E., Marini C., Lo T. W. B., Yang F., Zhang L., Liu L. (2025). Nat. Commun..

[cit36] Chen L., Verma P., Hou K., Qi Z., Zhang S., Liu Y.-S., Guo J., Stavila V., Allendorf M. D., Zheng L., Salmeron M., Prendergast D., Somorjai G. A., Su J. (2022). Nat. Commun..

[cit37] Wu J., Zhao G., Song M., Wang H., Wei Y., Chen X., Wang G., Yan Z. (2022). Fuel.

[cit38] Sukhbaatar B., Qing W., Seo J., Yoon S., Yoo B. (2024). Sci. Rep..

[cit39] Bhogeswararao S., Srinivas D. (2015). J. Catal..

[cit40] Betancourt P., Rives A., Hubaut R., Scott C. E., Goldwasser J. (1998). Appl. Catal., A.

[cit41] Zhang X., Guo A., Jiao S., Wang X., Wu J., Jia A., Zhang Z., Liu H., Liu Y., Wang Z. (2026). J. Colloid Interface Sci..

[cit42] Hu P., Zhang C., Chu M., Wang X., Wang L., Li Y., Yan T., Zhang L., Ding Z., Cao M., Xu P., Li Y., Cui Y., Zhang Q., Chen J., Chi L. (2024). J. Am. Chem. Soc..

[cit43] Barth R., Pitchai R., Anderson R. L., Verykios X. E. (1989). J. Catal..

[cit44] Chin S. Y., Williams C. T., Amiridis M. D. (2006). J. Phys. Chem. B.

[cit45] Deng W., Wang Y., Zhang S., Gupta K. M., Hülsey M. J., Asakura H., Liu L., Han Y., Karp E. M., Beckham G. T., Dyson P. J., Jiang J., Tanaka T., Wang Y., Yan N. (2018). Proc. Natl. Acad. Sci. U. S. A..

[cit46] Zhang L., Si R., Liu H., Chen N., Wang Q., Adair K., Wang Z., Chen J., Song Z., Li J., Banis M. N., Li R., Sham T.-K., Gu M., Liu L.-M., Botton G. A., Sun X. (2019). Nat. Commun..

[cit47] Adabi H., Shakouri A., Zitolo A., Asset T., Khan A., Bohannon J., Chattot R., Williams C., Jaouen F., Regalbuto J. R., Mustain W. E. (2023). Appl. Catal., B.

[cit48] Ojelade O. A., Zaman S. F. (2022). Arabian J. Sci. Eng..

[cit49] Zong Z., Rao C., Du C., Lu R., Upham D. C. (2025). Resour., Conserv. Recycl..

[cit50] Hernández B., Luo Y., Vlachos D., Ierapetritou M. (2026). ACS Sustainable Chem. Eng..

[cit51] Hernández B., Kots P., Selvam E., Vlachos D. G., Ierapetritou M. G. (2023). ACS Sustainable Chem. Eng..

[cit52] Zong Z., Daoud O., Hankins N. P., She Q., Peters C. D. (2025). Water Res..

[cit53] Lau W. W. Y., Shiran Y., Bailey R. M., Cook E., Stuchtey M. R., Koskella J., Velis C. A., Godfrey L., Boucher J., Murphy M. B., Thompson R. C., Jankowska E., Castillo Castillo A., Pilditch T. D., Dixon B., Koerselman L., Kosior E., Favoino E., Gutberlet J., Baulch S., Atreya M. E., Fischer D., He K. K., Petit M. M., Sumaila U. R., Neil E., Bernhofen M. V., Lawrence K., Palardy J. E. (2020). Science.

