# Supplementary material for: Methylcyclohexane-assisted polypropylene upcycling over PtRu/Al2O3via tandem dehydrogenation–hydrogenolysis
Source: Energy Environ Sci. 2026 Oct 27. Online ahead of print. doi: 10.1039/d6ee05130h (PMC13711158; doi:10.1039/d6ee05130h)
Supplement: Supplementary file 1 [file EE-OLF-D6EE05130H-s001.pdf]

Supplementary Information (SI) for Energy & Environmental Science.
This journal is © The Royal Society of Chemistry

Supplementary Information

Methylcyclohexane-assisted polypropylene upcycling over PtRu/Al_2O_3 via tandem dehydrogenation–hydrogenolysis

Haokun Wang[1#], Zhiyuan Zong[2#], Mingyu Luo[3#], Frederick Z.T. Yang[1], Jiaying Pan[2], Zeyu Wu[3], Rui Liu[3], Yuhong Luo[3], Jiasi Li[3], Yiyang Li[3], Roland C. Turnell-Ritson[1], Michail Stamatakis[3], Aidong Yang[2] and Dermot O' Hare[1*]

[1]Chemistry Research Laboratory, Department of Chemistry, University of Oxford, 12 Mansfield Road, Oxford OX1 3TA, United Kingdom

[2]Department of Engineering Science, University of Oxford, Parks Road, Oxford OX1 3PJ, United Kingdom

[3]Inorganic Chemistry Laboratory, University of Oxford, South Parks Road, Oxford OX1 3QR, United Kingdom

[#]Authors contributed equally: Haokun Wang, Zhiyuan Zong, Mingyu Luo

*Corresponding author: dermot.ohare@chem.ox.ac.uk

1. Supplementary Methods

1.1 Catalyst preparation

xPtyRu on g-Al_2O_3 catalysts with nominal Pt loadings of 0.5–2 wt% and Ru loadings of 0–5 wt% were prepared by a conventional co-impregnation method. In the manuscript, *x*Pt*y*Ru denotes the nominal synthesis composition in wt%; for example, PtRu refers to nanoparticulate catalyst prepared using 1 wt% Pt and 1 wt% Ru. The γ-Al_2O_3 support was prepared by calcining boehmite at 600 °C for 8 h in air. Chloroplatinic acid hydrate and ruthenium(III) chloride trihydrate were used as metal precursors and dissolved in deionised water prior to impregnation. The γ-Al_2O_3 support was then impregnated with the precursor solution under stirring at room temperature for 5 h. The resulting slurry was dried at 100 °C overnight and subsequently calcined in air at 550 °C for 6 h (heating rate: 5 °C min⁻^{1}). Prior to catalytic testing, the catalysts were reduced under a 5 vol% H_2/N_2 flow at 500 °C for 3 h. Other bimetallic catalysts (Pt3Ga/Al_2O_3, P2tNi/Al_2O_3, and Pt2Co/Al_2O_3) were synthesised following the same procedure using the corresponding metal precursors: gallium(III) nitrate hydrate, nickel(II) nitrate hexahydrate, and cobalt(II) nitrate hexahydrate. Monometallic Pt/Al_2O_3 and Ru/Al_2O_3 catalysts were prepared analogously.

1.2 Catalytic tests

PP hydrogenolysis assisted by MCH was conducted in a 50 mL stainless-steel Parr batch reactor. In a typical experiment, 2 g PP (Mw ~ 308 kDa), 0.2 g catalyst, and 0.02 mol MCH were loaded into the reactor unless otherwise specified. The reactor was purged three times and then charged with 3 bar helium. The reactor was heated to 300 °C over 60 min under stirring at 400 rpm using a glass-coated stirrer, and maintained at the target temperature for the desired reaction time. After completion, the reactor was cooled to room temperature prior to product analysis.

Commercial plastic samples were first cut into small pieces (~0.5 × 0.5 cm) using scissors. Subsequently, 2 g of the plastic was weighed and mixed with the catalyst and MCH solvent in the autoclave. All other reaction conditions were identical to those described previously (300 °C, 5 h, 0.02 mol MCH, 0.2 g catalyst, stirring at 400 rpm). All experiments exhibited mass balances within the range of 97 ± 3%.

Reusability tests were conducted under the same reaction conditions as described above, with all mass balances within 97 ± 3%. After each reaction, the spent catalyst was recovered from the autoclave as a liquid–solid mixture

using chloroform as the extraction solvent. The mixture was separated by centrifugation, and the recovered solid (spent catalyst) was dried at 100 °C overnight. The dried catalyst was then reused directly in subsequent reaction cycles without further treatment. Because some catalyst was inevitably lost during product separation, the amount of recovered spent catalyst used in each subsequent cycle was added to 0.2 g by supplementing it with catalyst recovered from other recycling runs.

Scaled-up experiments using 10 g PP were performed in the same autoclave under identical reaction conditions, with the only variation being the increased quantities of feedstock and catalyst (10 g PP, 1 g catalyst, and 0.1 mol MCH). All other conditions were maintained as before (300 °C, 5 h, stirring at 400 rpm), and all mass balances remained within 97 ± 3%.

1.3 Product analysis

For product analysis, gaseous products from the autoclave headspace were collected using a 250 μL gas-tight glass syringe and analysed by gas chromatography (GC) equipped with both a flame ionization detector (FID) and a thermal conductivity detector (TCD) for gas-phase composition determination. Liquid-phase products were analysed by gas chromatography–mass spectrometry (GC–MS, Agilent 6890), with chloroform used to extract liquid products from the solid–liquid mixture in the autoclave. Quantification of both gaseous and liquid products was carried out using a normalised method. Prior to analysis, the liquid–solid mixture was separated by centrifugation. The collected liquid phase was directly injected into the GC–MS for analysis, while the separated solid residue was dried at 100 °C overnight and subsequently weighed at room temperature.

The final products were classified into three phases: gas, liquid, and solid. The mass of the autoclave, including the stirrer, was initially measured using a balance with a capacity and accuracy of 10 kg ± 0.05 g. The mass of the liquid–solid mixture was determined from the difference between the weight of the autoclave after gas release and that of the empty autoclave (including the stirrer). The solid mass was obtained by subtracting the mass of the catalyst, and the liquid mass was subsequently calculated by subtracting the masses of the solid residue and catalyst from the total liquid–solid mixture.

The yield of a given product or solid could be calculated as:

$$\text{Yield [wt\%]} = \frac{m_i}{m_{gas} + m_{liquid} + m_{solid}} \times$$

Where m_i is the mass of a given product i, or solid, and m_{gas}, m_{liquid} and m_{solid} represent the masses of the gas, liquid and solid phases, respectively.

The selectivity of the product i was calculated as:

$$\text{Selectivity [wt\%]} = \frac{m_i}{\square\, m_i} \times 100\% \quad (2)$$

Where $\square\, m_i$ is the total mass of products in the gas and liquid phases (excluding solid residues).

Polymer conversion was defined as:

$$\text{Conversion [wt\%]} = \frac{m_{gas} + m_{liquid}}{m_{gas} + m_{liquid} + m_{solid}} \times 100\% \quad (3)$$

Mass balance was calculated as:

$$\text{Mass balance [\%]} = \frac{m_{gas} + m_{liquid} + m_{solid}}{m_{input}} \times 100\% \quad (4)$$

All reactions exhibited mass balances within 97 ± 3%. Because MCH is a reactive hydrogen donor rather than an inert solvent, the initially added solvent was excluded from the reported product distribution by deducting its mass from the corresponding carbon-number fraction. For example, when 0.02 mol MCH (1.96 g) was used, 1.96 g was subtracted from the C₇ fraction before calculating the final product distribution. This correction accounts for the initially introduced MCH; MCH-derived products such as toluene and other C₇ hydrocarbons were quantified separately where applicable.

1.4 Material characterisation

The crystal structures of the samples were characterised by powder X-ray diffraction (XRD) using a Bruker D8 Advance Eco diffractometer operated at 40 kV and 25 mA with Cu Kα₁ radiation.

Gel permeation chromatography (GPC) was performed using a high-temperature GPC system equipped with an IR5 infrared detector (GPC-IR5). Samples were prepared by dissolving polymers in 1,2,4-trichlorobenzene (TCB) containing ~300 ppm 3,5-di-tert-butyl-4-hydroxytoluene (BHT) at 160 °C for 90 min, followed by filtration through a 10 μm stainless steel filter. Measurements were conducted at a flow rate of 0.5 mL min⁻¹ using TCB

containing 300 ppm BHT as the mobile phase, with 1 mg mL^{-1} BHT added as a flow marker. The column and detector temperatures were maintained at 145 and 160 °C, respectively.

High-angle annular dark-field scanning transmission electron microscopy (HAADF-STEM) images were acquired using a JEOL JEM-2100 transmission electron microscope operating at 200 kV. Energy-dispersive X-ray spectroscopy (EDS) analysis was conducted with a detector providing a maximum count rate of 8 cps eV^{-1}, ensuring adequate resolution of all elemental signals.

Thermogravimetric analysis (TGA) was carried out using a TA Instruments SDT-Q600 system. Samples were stabilised under an air flow (20 mL min^{-1}) at room temperature for 10 min, after which the initial mass was recorded. The temperature was then increased to 700 °C at a heating rate of 10 °C min^{-1}.

Inductively coupled plasma mass spectrometry (ICP-MS) measurements were performed using a Thermo Fisher Scientific iCAP7400 instrument.

Hydrogen temperature-programmed reduction (H_2-TPR) experiments were conducted using a ChemBET Pulsar TPR/TPD system. Approximately 0.2 g of sample was loaded into a quartz reactor and pretreated at 300 °C for 1 h under argon flow. After cooling to 50 °C, the system was stabilised for 2 h under 5 vol% H_2/N_2 (20 mL min^{-1}). The temperature was then ramped to 600 °C at 5 °C min^{-1}, and hydrogen consumption was recorded.

X-ray photoelectron spectroscopy (XPS) was performed using a Thermo Scientific Nexsa spectrometer equipped with a monochromated Al Kα source (1486.7 eV), a spherical sector analyser, and multichannel detectors. Catalysts were pre-reduced under 5 vol% H_2/N_2 at 500°C for 3 h prior to analysis. Measurements were conducted under ultra-high vacuum (<10^{-8} Torr) at room temperature. Data were analysed using CasaXPS, with a Shirley background applied prior to peak deconvolution.

X-ray absorption spectroscopy (XAS) measurements were performed at beamline B18 of the Diamond Light Source. The Pt L₃-edge and Ru K-edge spectra were calibrated using Pt and Ru foil standards, respectively.

CO adsorption Fourier-transform infrared spectroscopy (CO-FTIR) was conducted in a vacuum cell using a Thermo Scientific Nicolet 6700 spectrometer equipped with ZnSe windows and a liquid nitrogen-cooled mercury cadmium telluride detector. Spectra were recorded with 128 scans at a resolution of 4 cm^{-1} and presented in absorbance units. Prior to measurement, samples were reduced under 5 vol% H_2/N_2 at 400 °C for 3

h. After cooling to room temperature, background spectra were collected under N_2 flow. CO was then introduced and allowed to adsorb for 1 h, followed by evacuation for 1 h to remove physiosorbed species. Spectra were subsequently recorded at room temperature.

1.5 Molecular dynamics simulations

Molecular dynamics (MD) simulations were conducted to investigate the solvent-dependent conformational behaviour of PP. A single PP chain comprising 35 repeat units ($C_{105}H_{212}$) was independently solvated in cyclohexane, methylcyclohexane, dimethylcyclohexane, decalin, dimethylpentane and heptane. Initial configurations were constructed with Packmol[1] by placing one PP chain and an appropriate number of solvent molecules, calculated from the experimental liquid density of each solvent, in cubic simulation boxes of 10 x 10 x 10 nm^3. Periodic boundary conditions were applied along all three Cartesian directions. Molecular interactions were represented using CHARMM general force-field parameters.[2]

The simulations were performed with LAMMPS 29 Aug 2024[3] using a 1 fs integration timestep. Bonded interactions were described by harmonic bond potentials together with CHARMM angle and dihedral terms. Non-bonded interactions were modelled using the CHARMM force-switching Lennard-Jones/Coulomb potential, with switching and cutoff distances of 10 and 12 Å, respectively. Long-range electrostatic interactions were evaluated using the particle-particle particle-mesh method[4] with a convergence criterion of 10^{-5}. Each system was first relaxed by conjugate-gradient energy minimization, followed by 10 ns of NPT equilibration at 574 K and 40 atm. Production simulations were then carried out for 10 ns in the NVT ensemble at 574 K. C-H bonds in the polymer were constrained using SHAKE algorithm,[5] whereas solvent molecules were propagated as rigid bodies during both equilibration and production. Coordinates from the production trajectories were recorded every 5 ps.

The conformational properties of PP in different solvents were analysed using MDAnalysis[6, 7] and quantified in terms of the end-to-end autocorrelation function, radius of gyration (Rg) and root mean square deviation (RMSD).

The end-to-end vector was defined as $R(t) = r_{end}(t) - r_{start}(t)$, where $r_{end}(t)$ and $r_{start}(t)$ denote the positions of the terminal carbon atoms of the PP chain. The corresponding normalised autocorrelation function was calculated according to the following equation:

$$e2e(\tau) = \frac{\langle R(\tau) \cdot R(0) \rangle}{\langle R(0) \cdot R(0) \rangle} \quad (5)$$

with angular brackets indicating an average over time origins. The relaxation time was taken as the value of τ at which $e2e(\tau)$ decreased to 1/e. The radius of gyration was calculated as follows:

$$Rg^2 = \left(\frac{1}{M}\right) \sum_i m_i \, |r_i - r_{cm}|^2 \quad (6)$$

where $M = \sum_i m_i$ and r_{cm} is the centre of mass of the polymer. RMSD was evaluated after optimal superposition of each PP conformation onto a reference structure, which defined as the average PP-chain conformation over the analysed production trajectory. It was calculated according to the following equation:

$$RMSD = \sqrt{\sum_i |r_i - r_{ref}|^2 / N} \quad (7)$$

where N is the number of PP atoms and r_{ref} is the corresponding reference coordinate.

1.6 Techno-economic analysis and life-cycle assessment

A combined techno-economic analysis (TEA), life-cycle assessment (LCA), and regional resource assessment was performed to evaluate the performance and scalability of the proposed plastic-to-naphtha conversion pathways. TEA was conducted using a discounted cash-flow framework to estimate the minimum selling price of naphtha under steady-state operation, while LCA quantified cradle-to-gate greenhouse gas emissions on a per-unit naphtha basis, including upstream feedstock production, process energy use, direct emissions, and associated credits from by-products and avoided processes. Process performance and economic outcomes were evaluated based on experimentally validated product yields and scaled using standard chemical engineering design correlations. In parallel, a country-level analysis was performed by integrating plastic waste availability, recycling rates, and naphtha demand to assess the potential contribution of plastic-derived naphtha across Europe. Detailed assumptions, input parameters, and calculation procedures are provided in the Supplementary Information.

1.6.1 Techno-economic analysis

The TEA was conducted using a discounted cash-flow model to estimate the minimum selling price (MSP) of Naphtha. The financial model assumes:

Plant lifetime (T): 20 years

10% Internal rate of return (IRR), with discounted cash flow (CF).

Net present value (NPV): 0 at MSP

$$NPV = 0 = \sum_{t=0}^{T} \frac{CF_t}{(1 + IRR)^t} \quad (8)$$

The base case plant capacity was considered at: 1,00 kg h^{-1} waste PP

Capital costs were scaled using the six-tenths rule, a common approximation for chemical process scale-up:

$$C_2 = C_1 \left(\frac{S_2}{S_1}\right)^{0.6} \quad (9)$$

where C is capital cost and S is plant capacity.[8]

Operating costs, including electricity, feedstocks, and carbon taxation—were assumed to scale linearly with plant throughput.

Cost categories:

The cost breakdown shown in Fig. 5b covers the following categories. The factor of each individual terms that toward the final cost of manufacturing was taken from the base case scenario in chapter 8 of the well-known chemical engineering handbook.[8]

The cost process flow diagrams and the heat integration networks are shown in Figure S15 and Tables S15-16.

Personnel costs

Operating labour: 4.5 labor per shift, $85000/labor regarding 2024 CEPCI value.[8, 9] Both cases require ~$1.1 million of labour cost.

CAPEX-related costs (an additional lang factor at 4.74 should be applied to the liquid-based plant)[10, 11]

Reaction vessel: $1,560,000 and 20 bar maximum pressure for MCH case. $3,060,000 and 40 bar maximum pressure for H_2 case. (150 m^3 reflects 6 h reaction residence time and made with stainless steel for both cases)[8]

Combustion vessel: $23,100 and $33,600 for MCH and H_2 cases, respectively, which reflects ~ 1s combustion residence time[8]. Both are 10 bar maximum allowed pressure and made with Nickel Clad material.

Distillation columns: \$127,700 and \$118,500 for MCH and H_2 cases, respectively (Aspen)

Heat exchange network: \$131,100 and \$107,900 for MCH and H_2 cases, respectively (Aspen Energy Analyzer)

3-phase separator: \$24,700 and \$22,700 for MCH and H_2 cases, respectively (Aspen)

Pumps: \$11,500 and \$66,800 for MCH and H2 cases, respectively. H_2 case was operated under 30 bars, which has higher demand on the pressurize devices[8]

Total equipment costs: \$1.88M and \$3.41M for MCH and H_2 cases. The cost breakdown is shown in Figure S16. Despite the high unit price of Ru, its contribution to total production cost is limited by the low catalyst loading; the 1,000 kg h^{-1} base-case plant contains approximately 1 kg Ru, corresponding to ∼US\$20,000 $year^{-1}$ under a conservative annual-replacement assumption.

OPEX-related costs

Feedstocks and electricity:

Grey hydrogen: \$2/kg[12]

MCH: \$966/t. The data was taken from the big commercial chemical trading website to show the relatively steady wholesale price as the benchmark.[13]

Waste PP: \$90/t for collecting and transfer cost. This cost estimation represents the lower bound from high income countries and upper range from the upper-middle income countries.[14]

Electricity consumption: 559 and 216 MWh per year by pumps and distillation column[15] for MCH and H_2 cases, respectively, with the rate at \$0.1/kWh.[16] The reboilers at the distillation column were assumed to be electricity driven.

Policy-related costs

Carbon tax: \$60/t-CO_2 for base case 45Q rate.[17] The direct emissions from tail gas combustion result in 349 and 883 kg/h of CO_2e for MCH and H_2 cases, respectively.

By-product revenues

Diesel: \$830/t-diesel. The data was taken from the big commercial chemical trading website to show the relatively steady wholesale price as the benchmark.[13]

Solid carbon black: \$150/t-carbon

Steam generation: excess heat from heat integration network and tail gas combustion would be used to generate steam for extra revenue. MCH and H_2 cases generate ~\$65,000 and ~\$191,000 revenue from steam generation, respectively.

Avoided polyethylene end-of-life (EoL) treatment: the minimum EoL landfill tipping cost in the US would be \$45/t-waste.[18]

Land cost: 10% of the CAPEX was assumed to be constant across plant scales.

Sensitivity analysis

The operating costs such as raw materials and utility cost are scaled proportionally to match the manufacturing demand. The capital cost is scaled using the scaling equation that is described in section A, whereas the land cost is assumed to remain the same.

A waste-PP throughput of 1,000 kg h^{-1} was adopted as the reference capacity, with 100–5,000 kg h^{-1} examined in the sensitivity analysis. This range is consistent with scales previously considered in techno-economic assessments of plastic conversion. Fivga and Dimitriou evaluated throughputs from 100 kg h^{-1} to 1,000 kg h^{-1} and above,[19] Jiang et al. considered 500–2,000 kg h^{-1}, and Riedewald et al. assessed a 40,000 t $year^{-1}$ facility, equivalent to approximately 5,333 kg h^{-1} at 7,500 operating hours per year.[20,21] These values are used only to contextualize the selected assessment range. The present analysis remains prospective, with experimentally determined yields and selectivities retained as scale-up assumptions.

1.6.2 Life-cycle assessment

The LCA evaluates cradle-to-gate greenhouse gas emissions associated with naphtha production.

The CO_2 emissions sector that included in the system boundary:

- hydrogen and MCH production: Grey and green hydrogen emissions are 9 and 0.88 kg CO_2e per kg H_2, respectively. The carbon intensity of conventional MCH is 3.73 kg CO_2e per kg MCH. Because a robust

route-specific life-cycle carbon intensity for bio-based MCH is currently unavailable, a proxy scenario was constructed by assuming the same upstream production burden as conventional MCH while treating the carbon contained in MCH as biogenic. On a stoichiometric basis, this biogenic carbon corresponds to 3.14 kg CO_2 per kg MCH, resulting in a proxy carbon intensity of 0.59 kg CO_2e per kg MCH. This value is therefore used as a low-carbon reference rather than a definitive carbon intensity for bio-based MCH, with 0.59–3.73 kg CO_2e per kg MCH representing the assumed interval between the bio-based proxy and the conventional fossil-MCH benchmark.

- plastic collection and sorting: 44 kg-CO_2e/ton-plastic[22]
- electricity consumption: 0.24 kg-CO_2e/kWh (projected share of 2030 mixed grid with current carbon intensity of each generation source)[23, 24]
- cooling utilities: 0.0000503 kg/kJ (Aspen, US NG based on EPA-title 40)
- direct process emissions: The direct emissions from tail gas combustion result in 349 and 883 kg/h of CO_2e for MCH and H_2 cases, respectively.

The CO_2 credits that are included in the system boundary:

- Steam generation credit: 0.0000503 kg/kJ (Aspen, US NG based on EPA-title 40). Heat generated from the exothermic reaction and combustion of tail gases is recovered as steam. This steam is assumed to displace natural-gas-derived steam generation. Regional variation in the associated emission credit reflects differences in the life-cycle carbon intensity of natural gas supply chains and will be explained in the next section.
- Avoided plastic end-of-life emissions. Because waste polypropylene is diverted from conventional disposal pathways, emissions associated with landfill, recycling, and incineration are avoided. The avoided-emission credit was calculated using a weighted global distribution of plastic waste treatment pathways, i.e., 49% landfilled, 22% unmanaged, 19% incineration, and 9% recycled, based on published global plastic waste statistics.[25]

The detailed share (in %) of EoL treatment methods regarding landfill, incineration, recycling and unmanaged waste can be found at OECD database.[25] The corresponding CO_2 intensity for each method are 0.08, 2.73 and 0.348 for calculating the weighted CO_2 emission.[22] The recycled option also avoided the fresh fossil-based plastic

production and reduced the additional ~3 kg-CO_2e/kg-PE and ended up with around -2.5 and -2.8 kg-CO_2eper kg PP or PE, respectively. Although unmanaged waste poses significant environmental risks, including leakage and ecosystem impacts, its contribution to direct greenhouse gas emissions is low, due to the persistence of plastics in the environment. The weighted end-of-life (EoL) emissions, based on the global average, are approximately 0.3 kg CO_2e per kg of plastic. This aligns with Organization for Economic Co-operation and Development (OECD) findings, which indicate that EoL emissions account for roughly 10% of those from the production stage.[26]

Byproducts: The emission avoided by the byproducts include diesel (0.888 kg-CO_2e/kg-diesel) and carbon coke (0.677 kg-CO_2e/kg-coke), taken from Ecoinvent database GLO market scenario.[24]

1.6.3 Regional sensitivity analysis and resource availability

Potential capacity of waste plastic

Country-level plastic waste generation was obtained from Eurostat.[27] To estimate the fraction of waste suitable for chemical upcycling, national recycling rates were applied to total plastic waste generation for each country.[28] This approach assumes that only plastics entering formal recycling streams are accessible as feedstock, while plastics disposed of via landfill, incineration, or uncontrolled dumping are excluded due to technical and logistical constraints.

The resulting recyclable plastic quantity represents the maximum available feedstock for plastic-to-naphtha conversion under current waste management systems.

Substantial variation in recyclable plastic availability was observed across Europe, reflecting differences in both total plastic consumption and recycling performance. Countries with high waste generation and strong recycling systems (e.g., Germany, Spain, Italy) exhibit the largest absolute feedstock potential. In contrast, countries with lower recycling rates or smaller waste streams show limited feedstock availability, constraining their potential contribution to plastic-derived naphtha production.

Potential production of plastic-based Naphtha

The potential production of naphtha from recycled plastics was estimated to be using a process yield derived from experimental and process modeling data. Specifically, 1000 kg of waste PP was assumed to produce 1092 kg of product classified as naphtha. The calculated naphtha production potential scales directly with available

recyclable plastic feedstock. Countries with high recycling volumes translate this into substantial theoretical naphtha production capacity.

To ensure conservative estimates, unreacted MCH present in the product stream was excluded from the net naphtha yield, despite being co-produced and potentially marketable. The exclusion of unreacted intermediates ensures that reported values reflect conservative, market-ready naphtha equivalents rather than total liquid product yield.

While the baseline assessment assumes all recycled plastics are available as feedstock, PP constitutes only 18.5% of the total plastic waste stream. Accordingly, when PP is considered as the sole feedstock, the estimated country-level naphtha production should be scaled by 0.185.[23] This demonstrates the importance of mixed plastic streams and feedstock flexibility for maximizing system performance.

Regional Naphtha demand and plastic upcycling intentionality

The estimated plastic-derived naphtha production was compared against country-level naphtha consumption to evaluate its relative contribution.[29] Results are expressed as the percentage of domestic naphtha demand that could be met through plastic upcycling.

Based on this comparison, countries were classified into three categories:

- Import-dependent countries (bold and underlined)

These countries have insufficient domestic naphtha production to meet demand and rely on imports.

In these regions (e.g., Spain, Sweden, Poland), plastic-derived naphtha represents a strategically significant resource. Even partial substitution could:

- Reduce import dependency
- Enhance supply security
- Improve circularity within domestic petrochemical systems

Plastic upcycling in these countries is therefore not only environmentally beneficial but also economically and geopolitically relevant.

- Self-sufficient countries (blue, non-bold)

These countries produce sufficient naphtha domestically to meet demand.

In these cases, plastic-derived naphtha functions primarily as a supplementary or alternative feedstock rather than a necessity. Its adoption will likely depend on:

- Cost competitiveness
- Policy incentives
- Decarbonization strategies

Thus, implementation is more sensitive to market conditions than supply constraints.

- Surplus-potential countries (yellow; >100% of demand)

These countries exhibit plastic-derived naphtha production potential exceeding their domestic consumption.

This outcome indicates that plastic waste streams in these countries could support net-export-oriented circular carbon systems. Specifically:

- Excess production could be exported to neighboring regions with naphtha deficits
- These countries could act as regional hubs for plastic upcycling and secondary feedstock supply
- Alternatively, surplus capacity could be redirected toward other petrochemical intermediates or fuels

Importantly, this also suggests a structural mismatch between waste availability and local petrochemical demand, highlighting opportunities for cross-border integration of circular supply chains.

2. Supplementary Figures

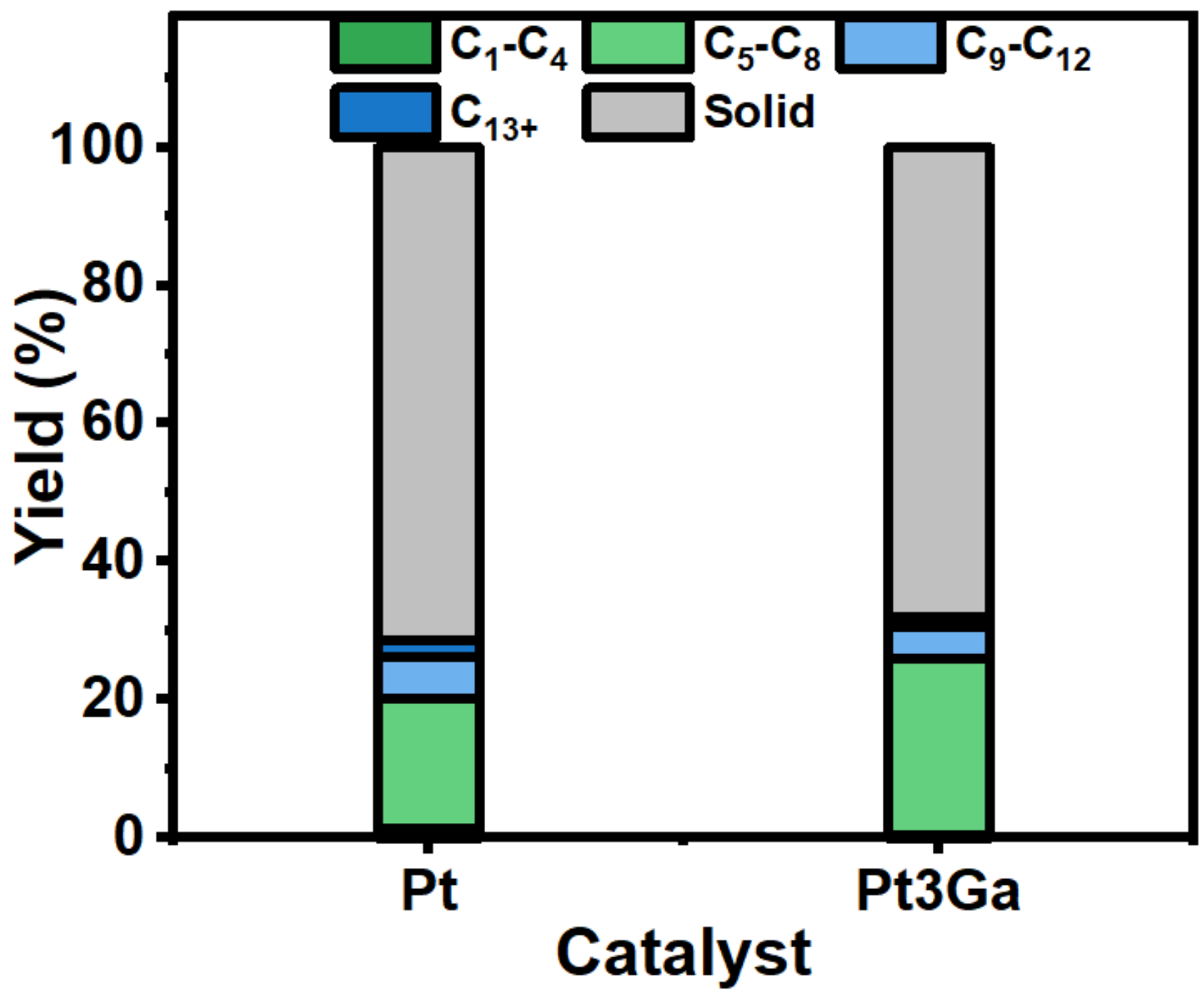

Supplementary Figure 1. Catalytic performance of Pt/Al_2O_3 and $Pt3Ga/Al_2O_3$. Reaction conditions: 2 g PP (Mw~308 kDa), 0.2 g catalyst, 300 °C, 5 h, 0.02 mol MCH, stirring at 400 RPM, 3 bar He.

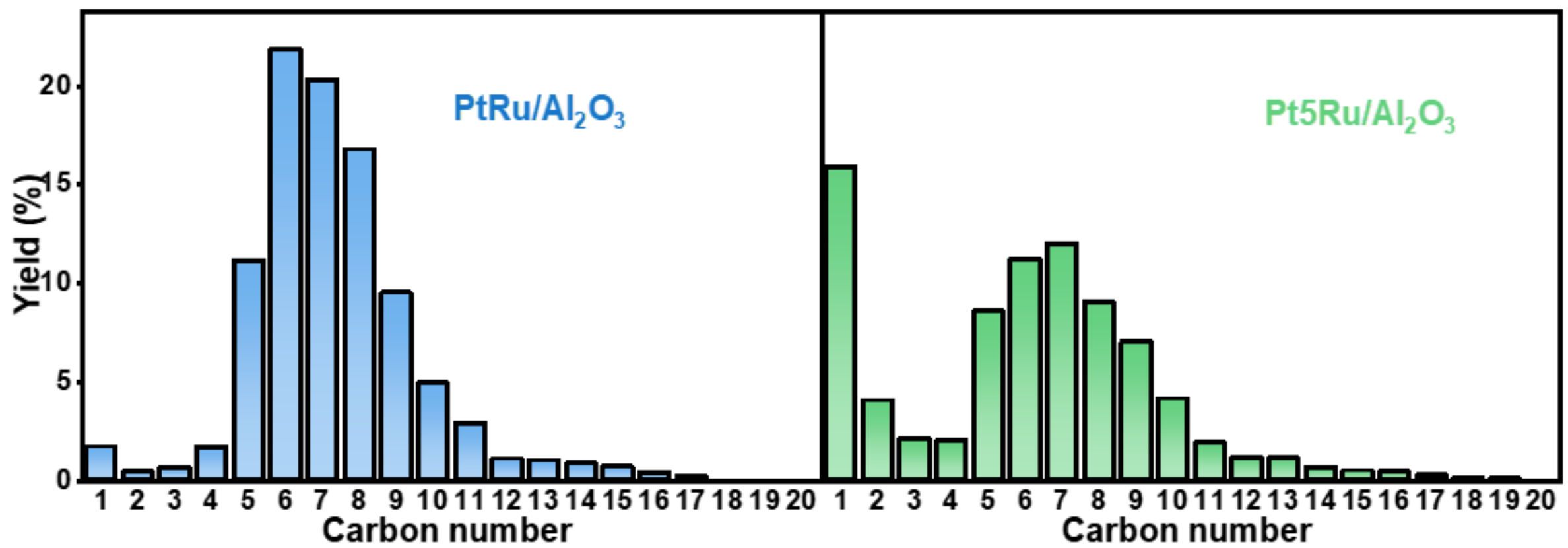

Supplementary Figure 2. Detailed carbon-number distribution over PtRu/Al_2O_3 and Pt5Ru/Al_2O_3. Reaction conditions: 2 g PP (Mw∼308 kDa), 0.2 g catalyst, 300 °C, 5 h, 0.02 mol MCH, stirring at 400 RPM, 3 bar He.

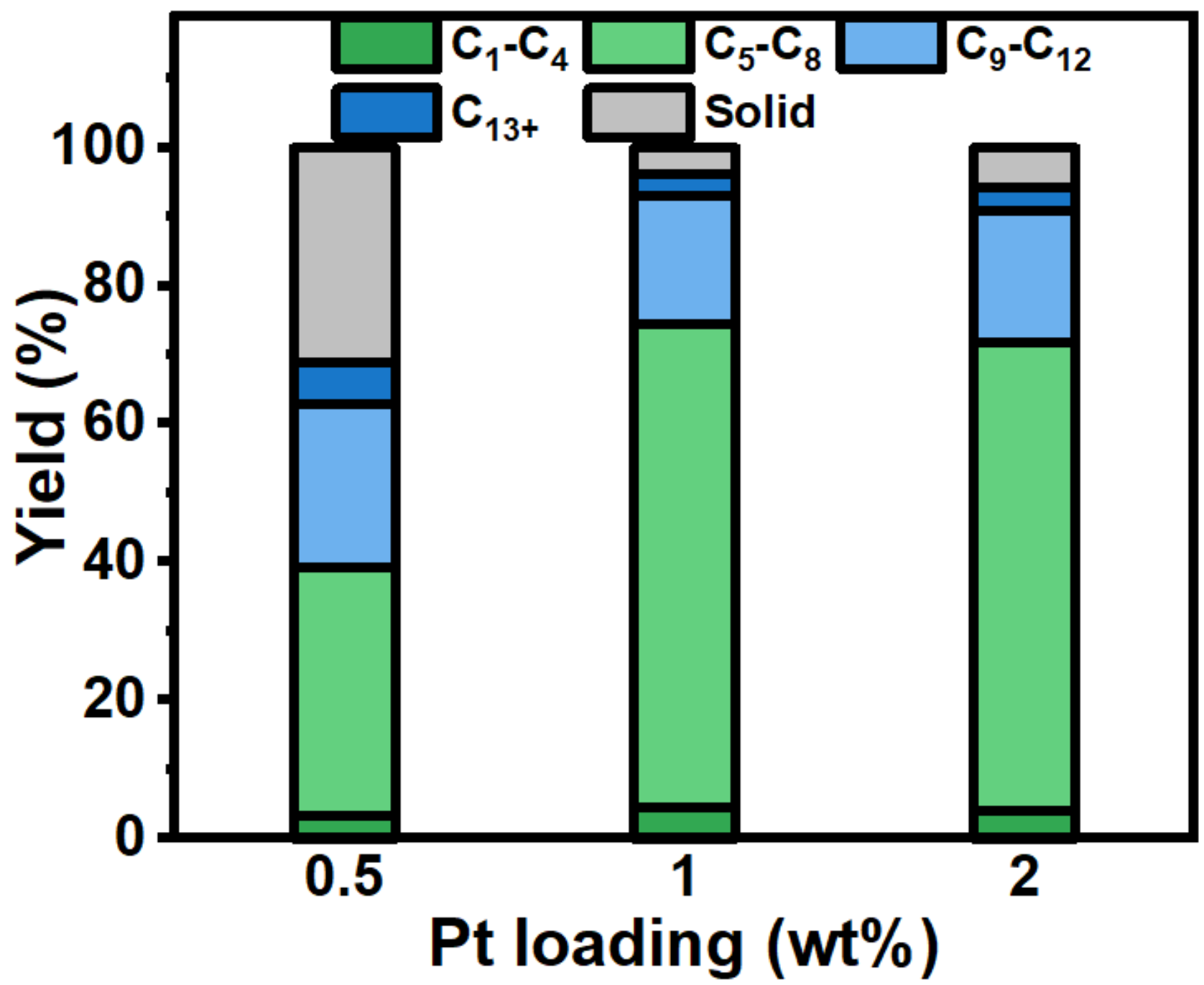

Supplementary Figure 3. Catalytic performance of xPtRu/Al₂O₃ catalysts with different Pt loadings. The Pt loading was varied from 0.5 to 2 wt%, while the Ru loading was fixed at 1 wt%. Reaction conditions: 2 g PP (Mw ~ 308 kDa), 0.2 g catalyst, 0.02 mol MCH, 300 °C, 5 h, stirring at 400 rpm, and 3 bar He.

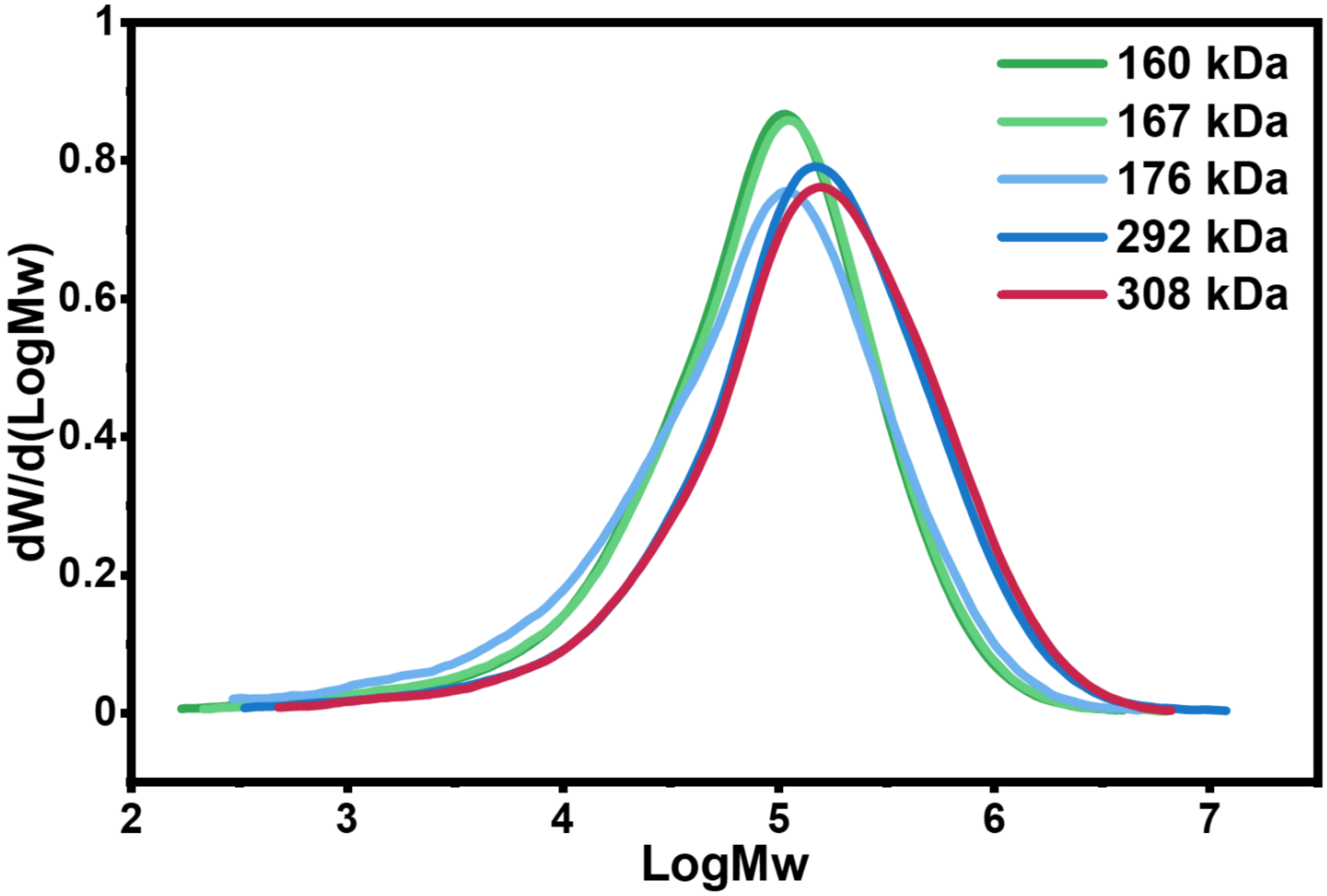

Supplementary Figure 4. GPC results for different molecular weight PP.

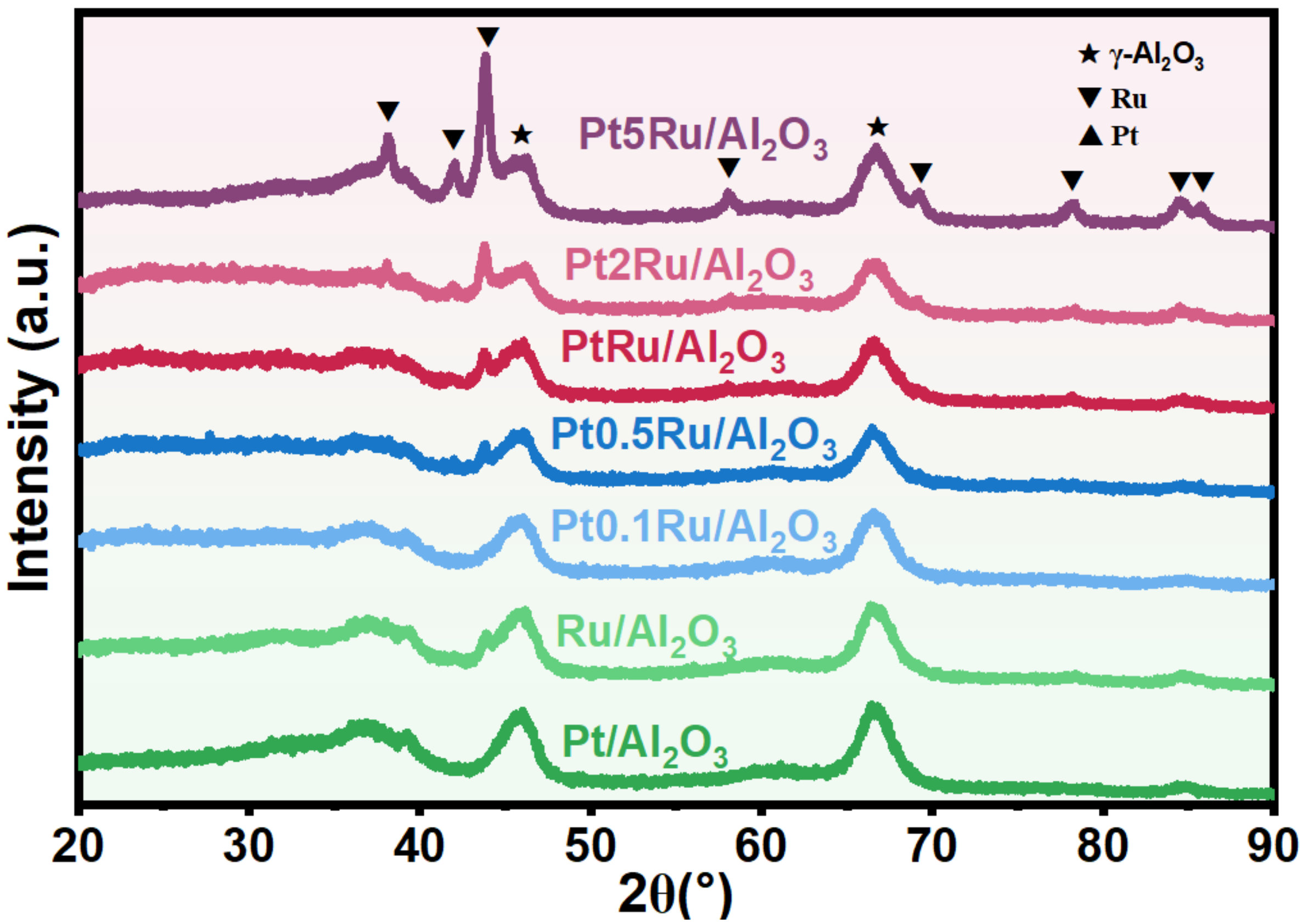

Supplementary Figure 5. XRD patterns for different $PtRu/Al_2O_3$ catalysts.

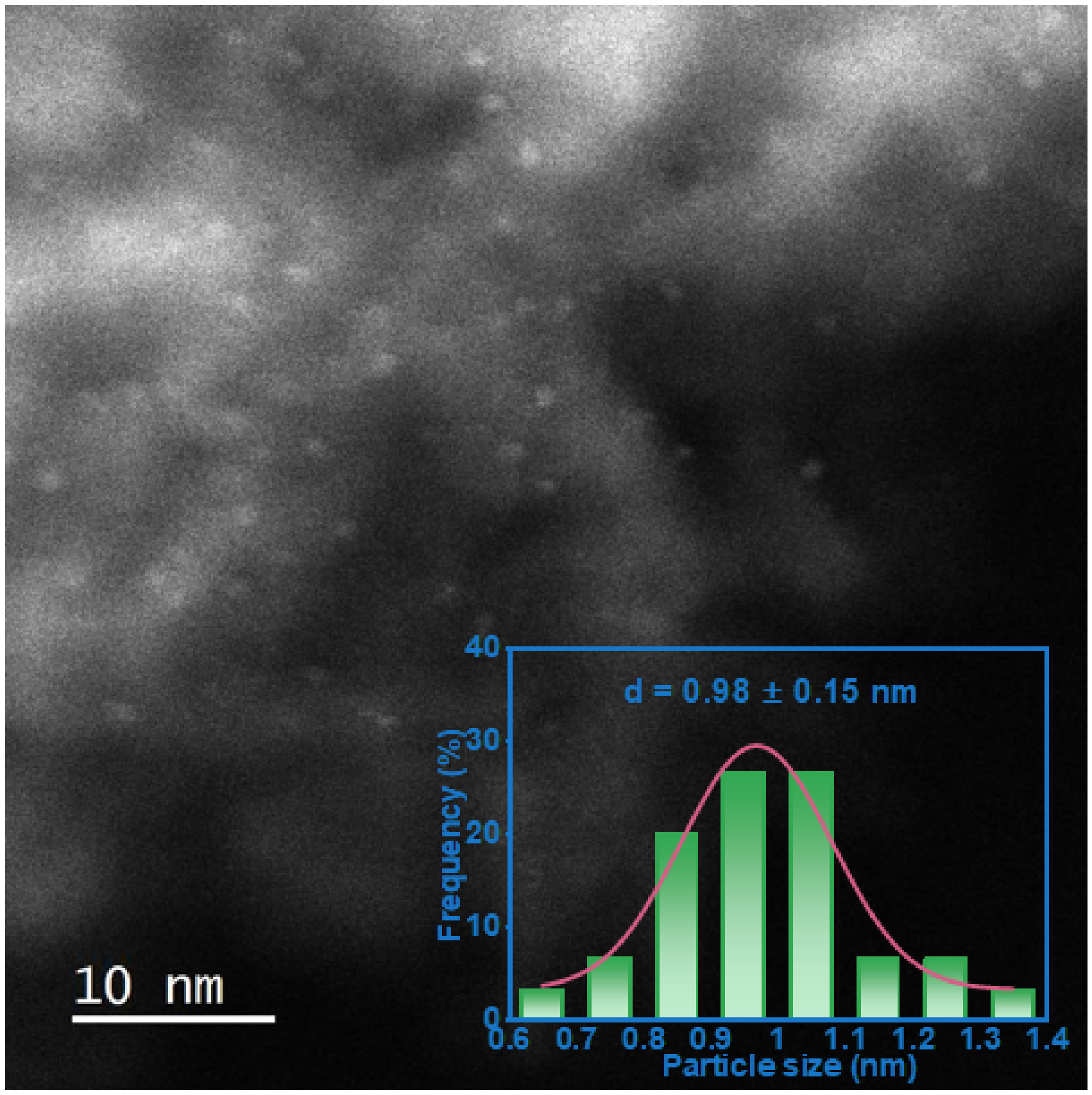

Supplementary Figure 6. HAADF-STEM image for $PtRu/Al_2O_3$ catalyst with particle size distribution.

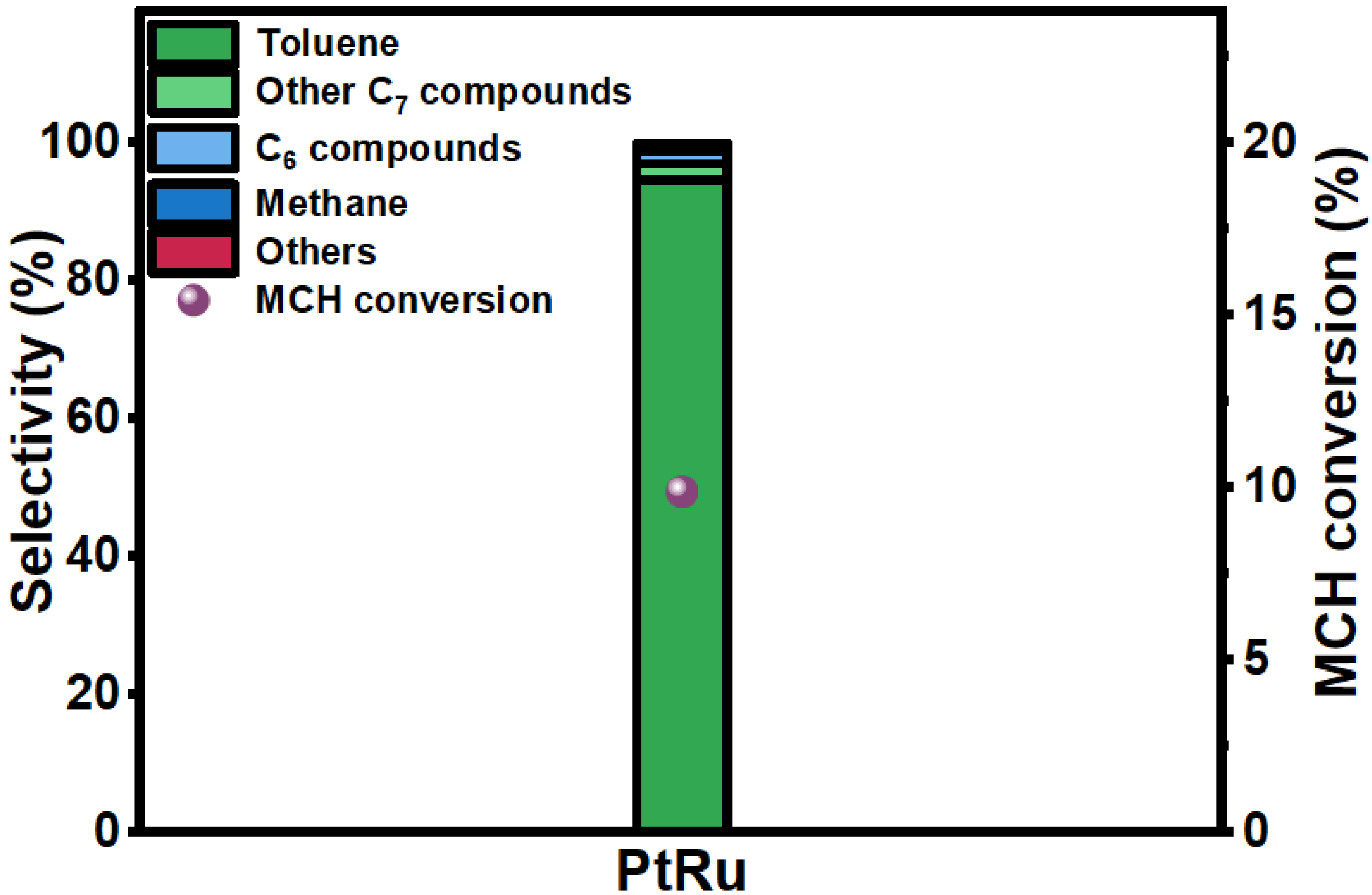

Supplementary Figure 7. Catalytic performance of MCH dehydrogenation without PP addition over PtRu/Al_2O_3. Reaction conditions: 0.02 mol MCH, 0.2 g PtRu/Al_2O_3, 300 °C, 5 h, stirring at 400 RPM, 3 bar He.

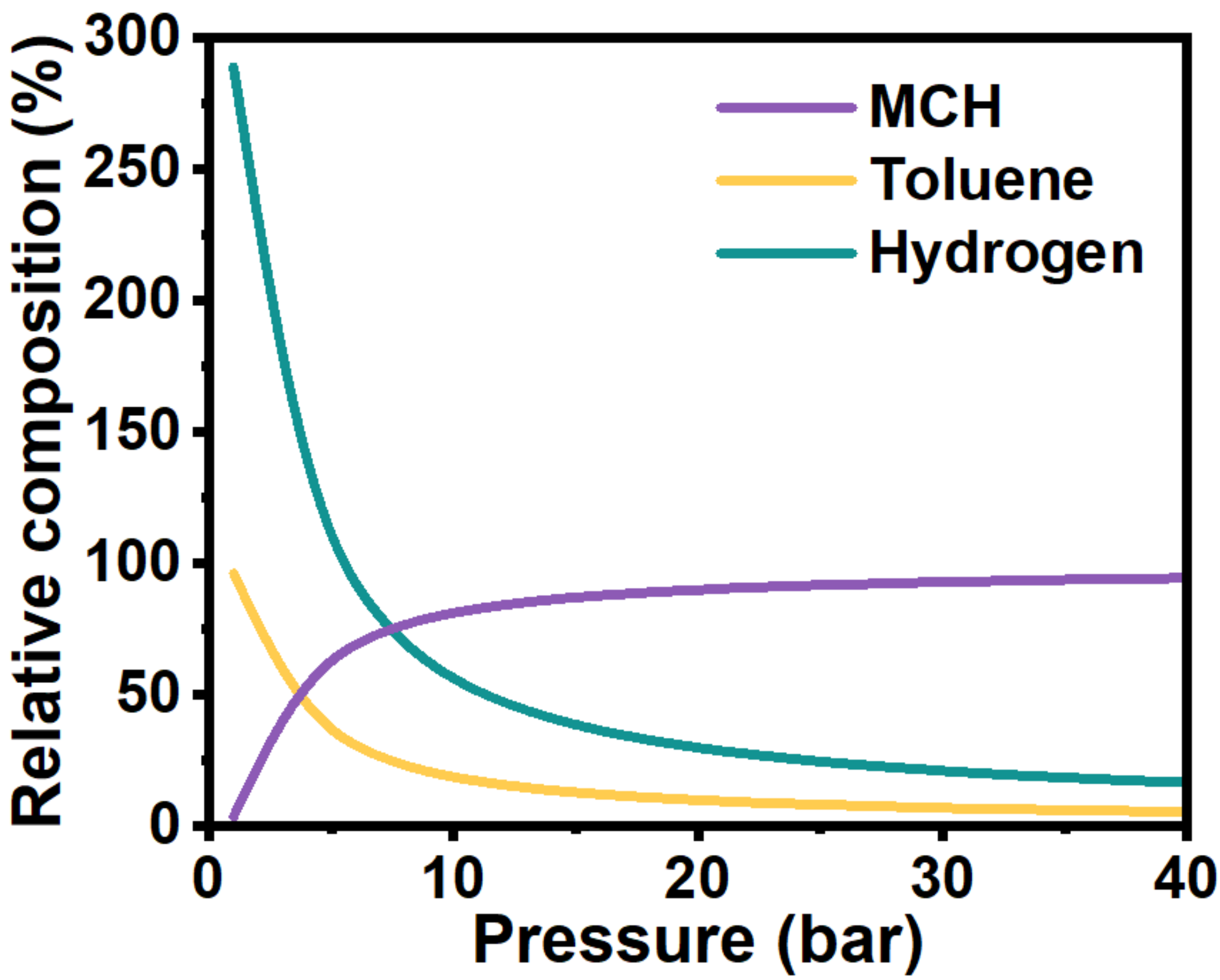

Supplementary Figure 8. Equilibrium composition of MCH dehydrogenation reaction at 300 °C as a function of different pressure.

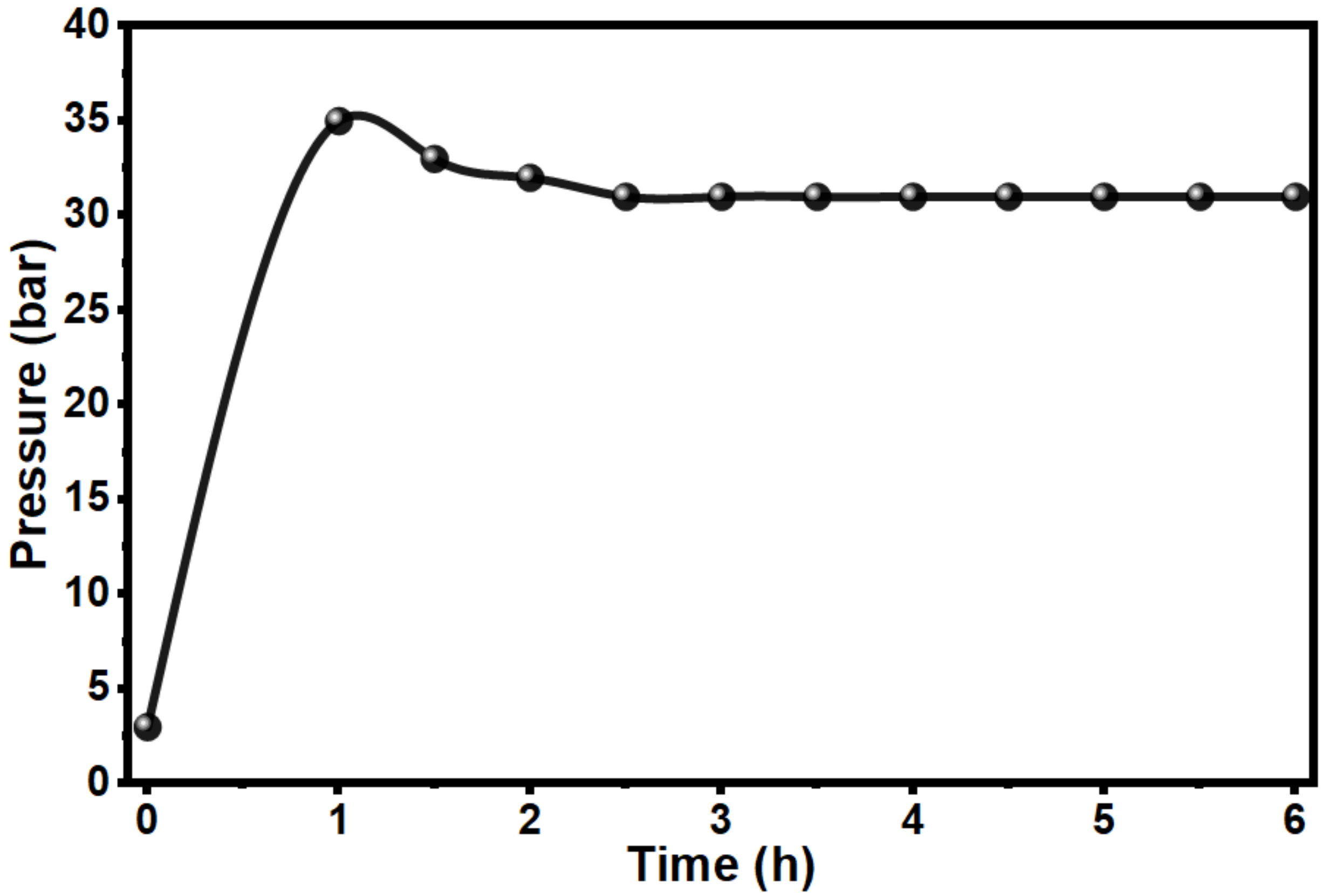

Supplementary Figure 9. Autoclave pressure profile during the reaction. Pressure evolution in the autoclave over the whole experiment. The initial period (0–1 h) corresponds to the heating stage from room temperature to 300 °C, followed by the reaction stage (1–6 h) under isothermal conditions.

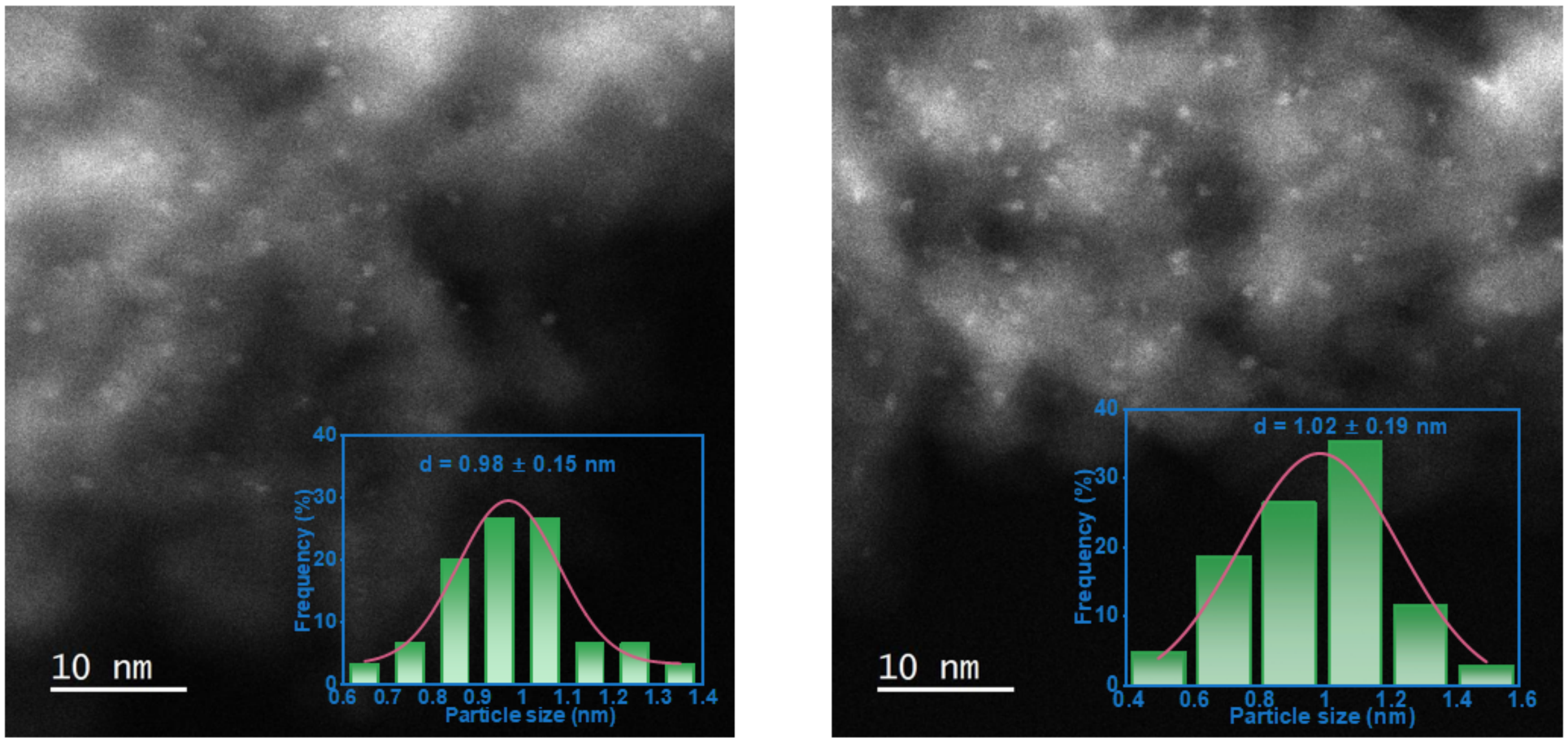

Supplementary Figure 10. HAADF-STEM image for fresh $PtRu/Al_2O_3$ (left) and spent $PtRu/Al_2O_3$ after 5th cycle with particle size distribution.

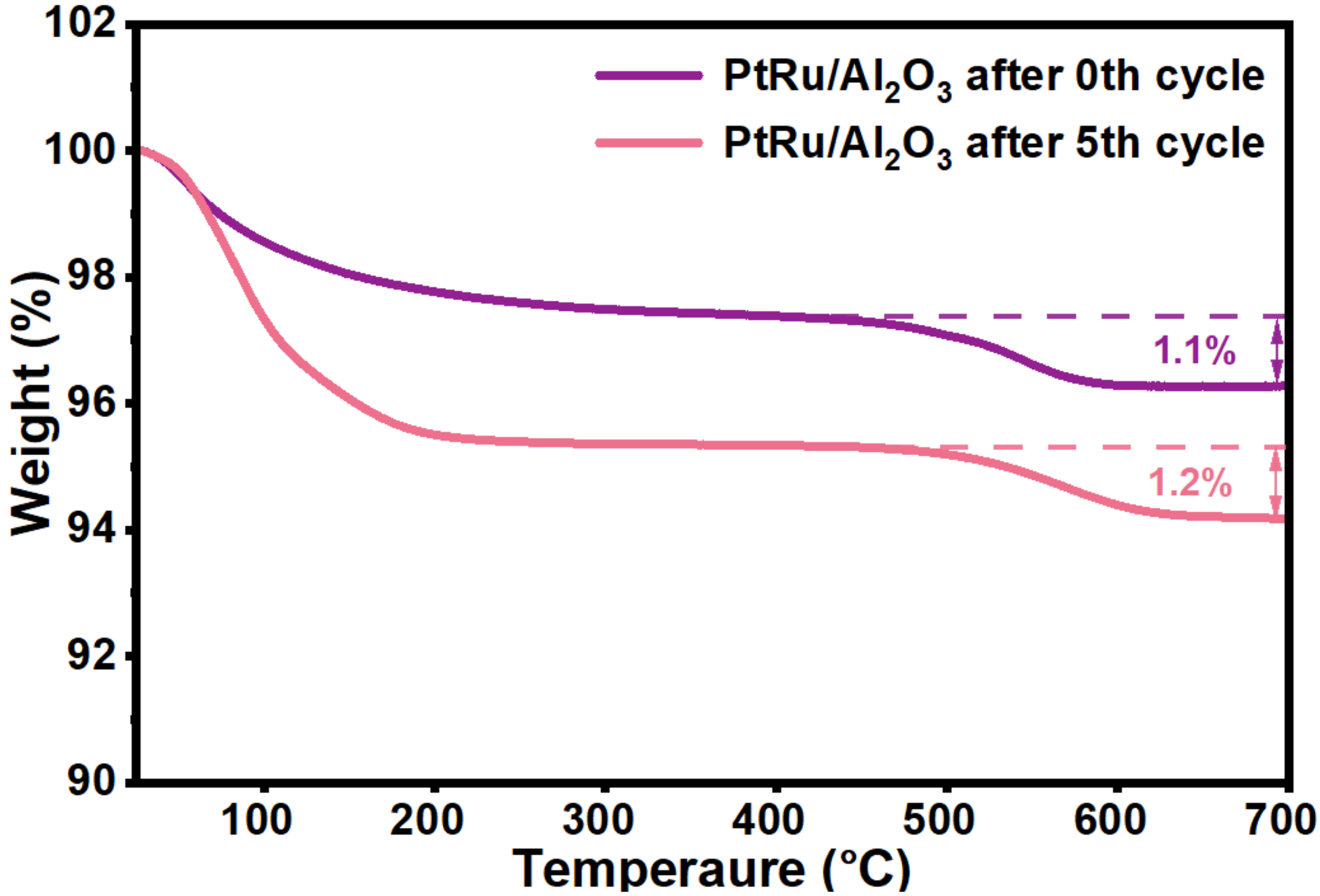

Supplementary Figure 11. TGA for spent PtRu/Al_2O_3 catalyst after 0th cycle and 5th cycle.

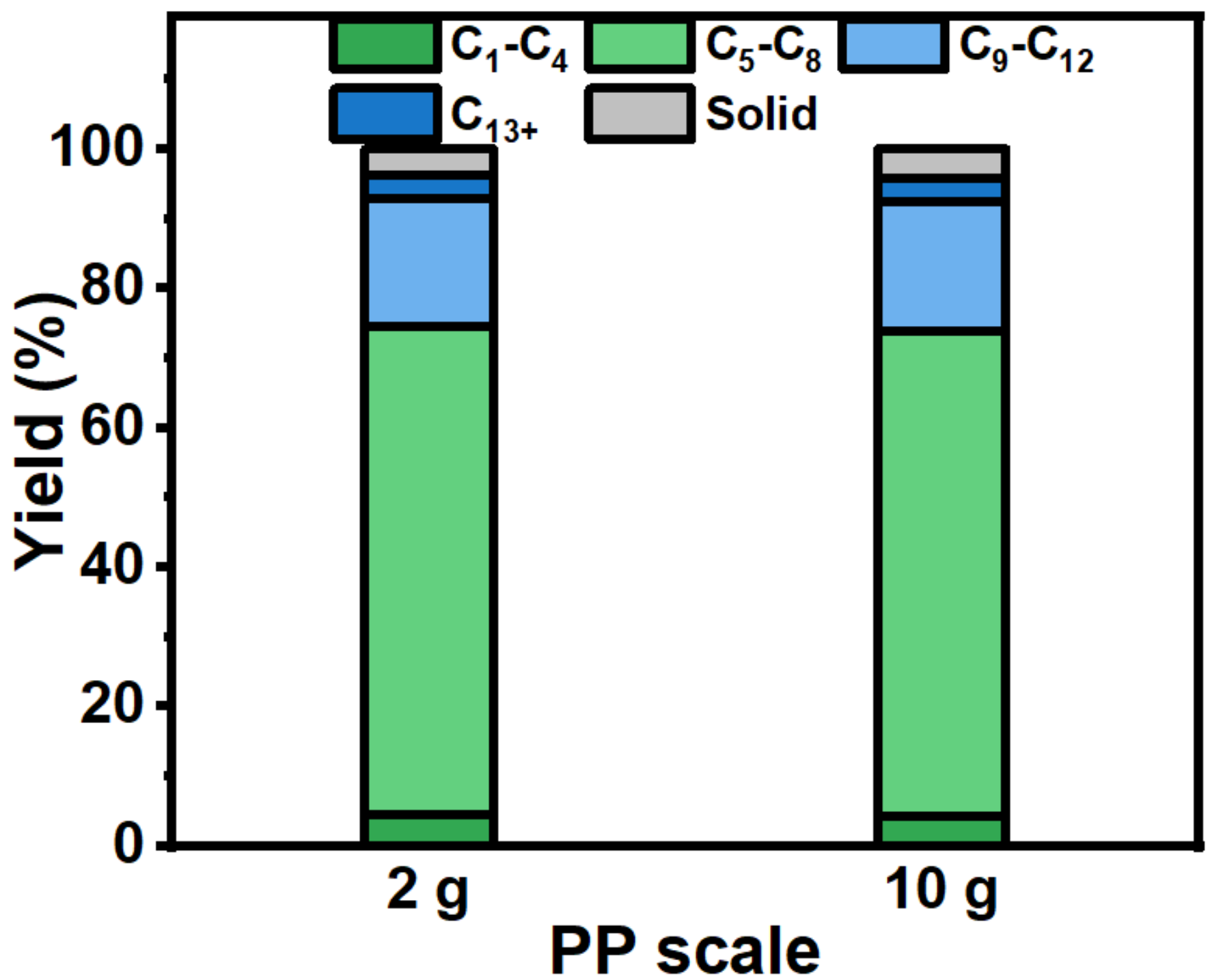

Supplementary Figure 12. Catalytic performance at different PP scales. Reaction conditions: 2 g or 10 g PP (Mw~308 kDa), 0.2 g or 1.0 g PtRu/Al_2O_3 catalyst, 300 °C, 5 h, 0.02 mol or 0.1 mol MCH, stirring at 400 RPM, 3 bar He.

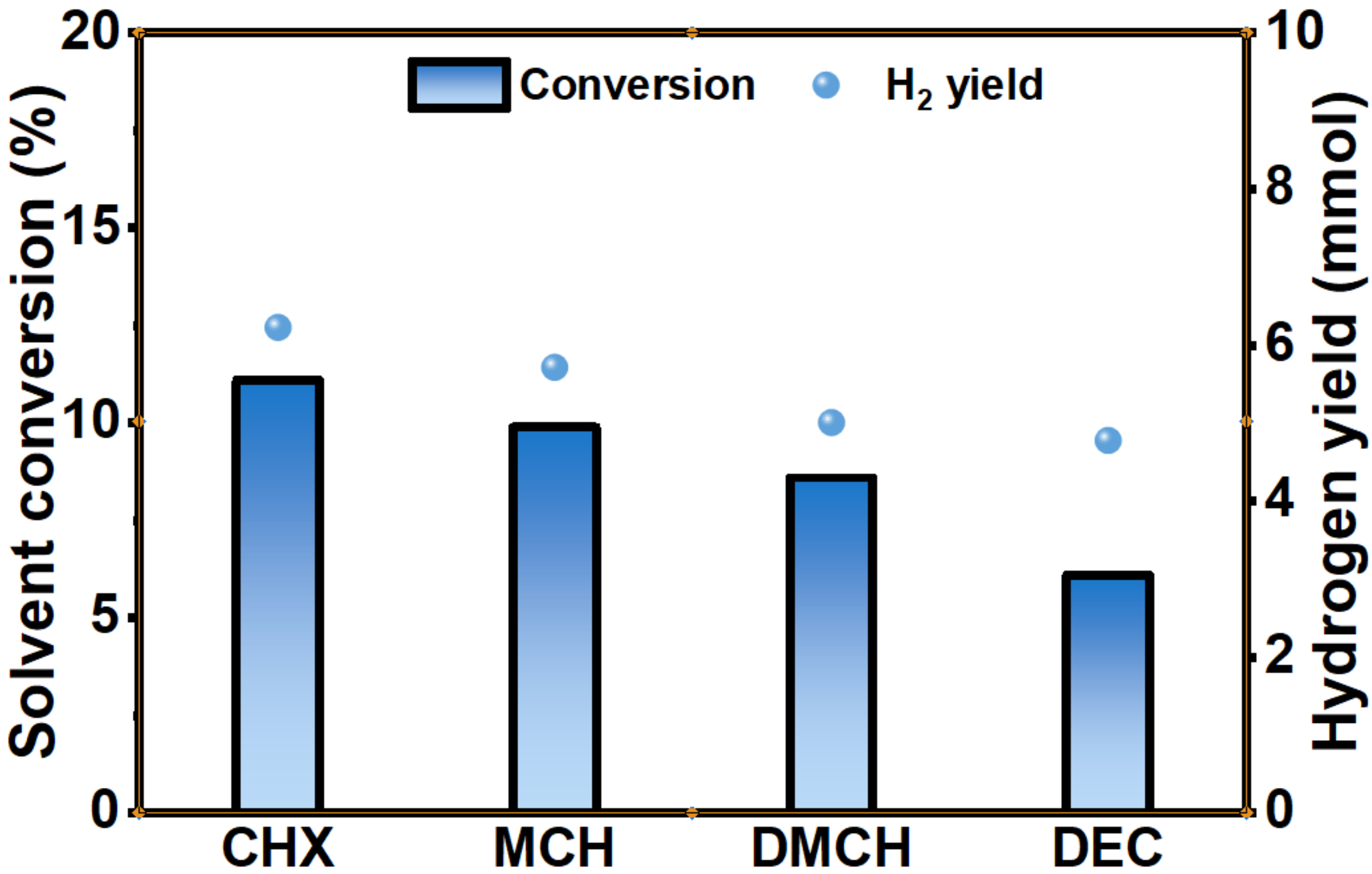

Supplementary Figure 13. Solvent conversion and hydrogen production using different LOHCs without PP. Reaction conditions: 0.2 g PtRu/Al_2O_3 catalyst, 300 °C, 5 h, 0.02 mol LOHC, stirring at 400 RPM, 3 bar He.

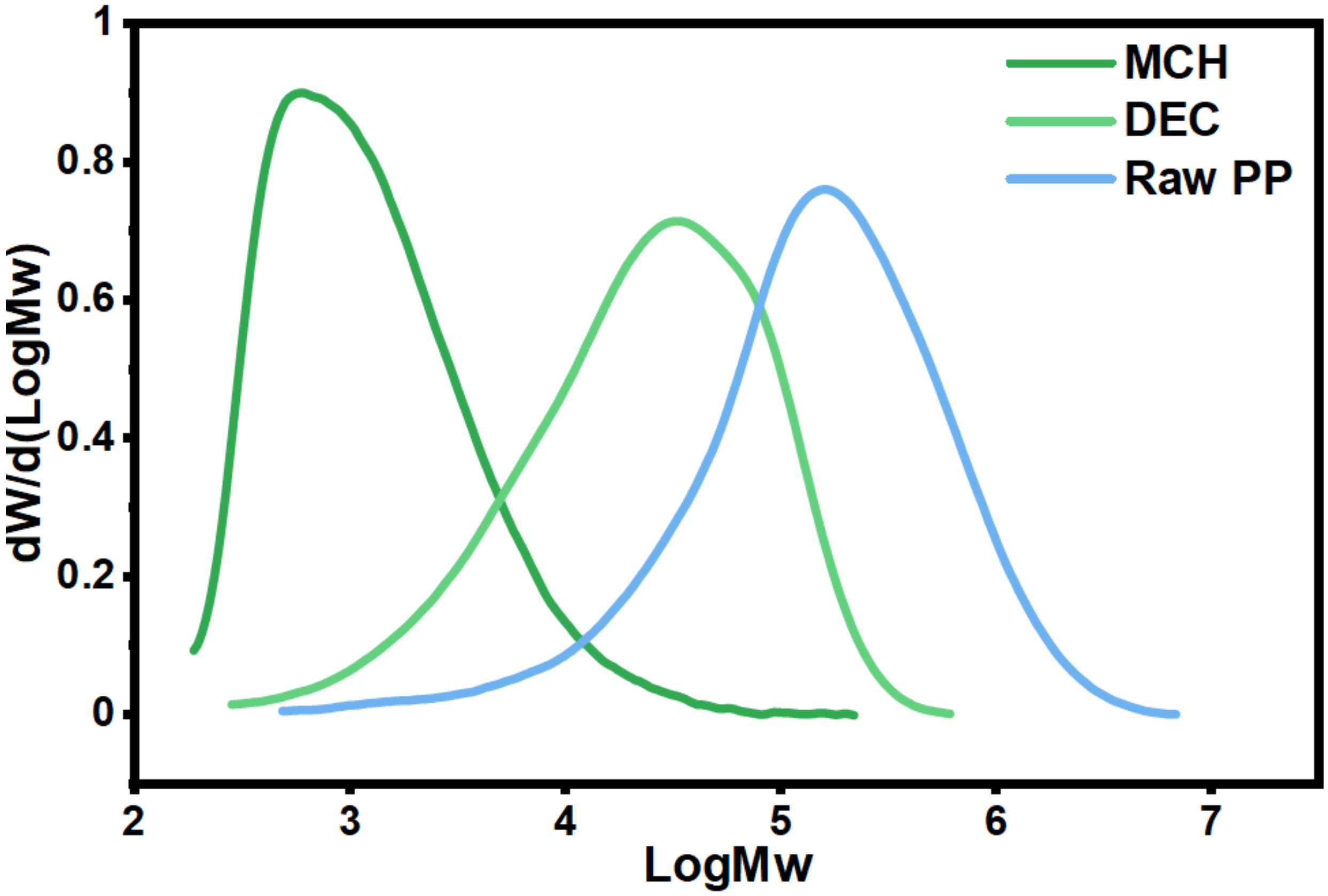

Supplementary Figure 14. GPC results of solid residues over different LOHCs. Reaction conditions: 2 g PP (Mw∼308 kDa), 0.2 g PtRu/Al_2O_3 catalyst, 300 °C, 5 h, 0.02 mol MCH/DEC, stirring at 400 RPM, 3 bar He.

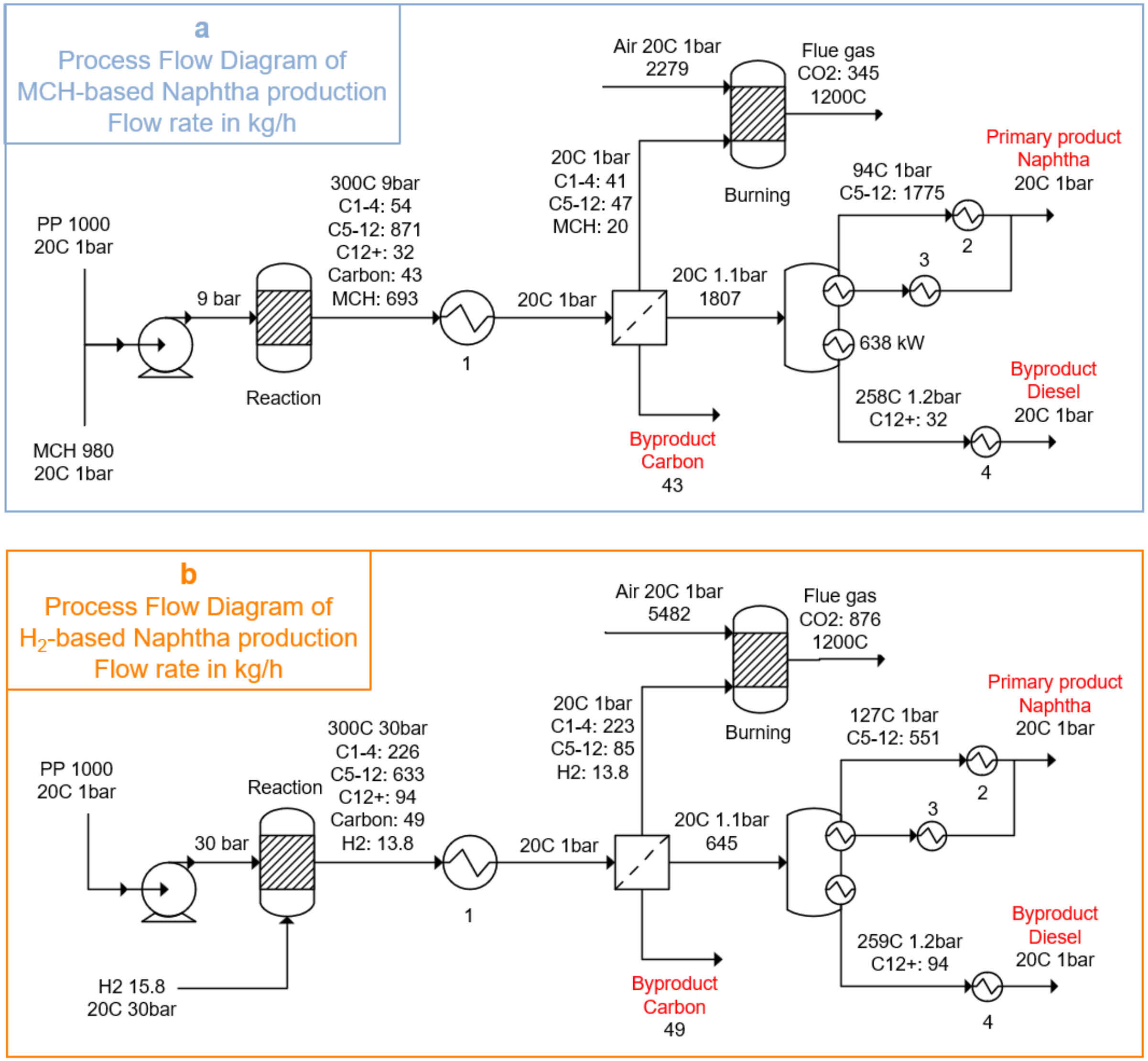

Supplementary Figure 15. Process flow diagrams of (a) MCH-based and (b) H_2-based naphtha production. The heat integration network is presented in the supplementary table 15-16 below.

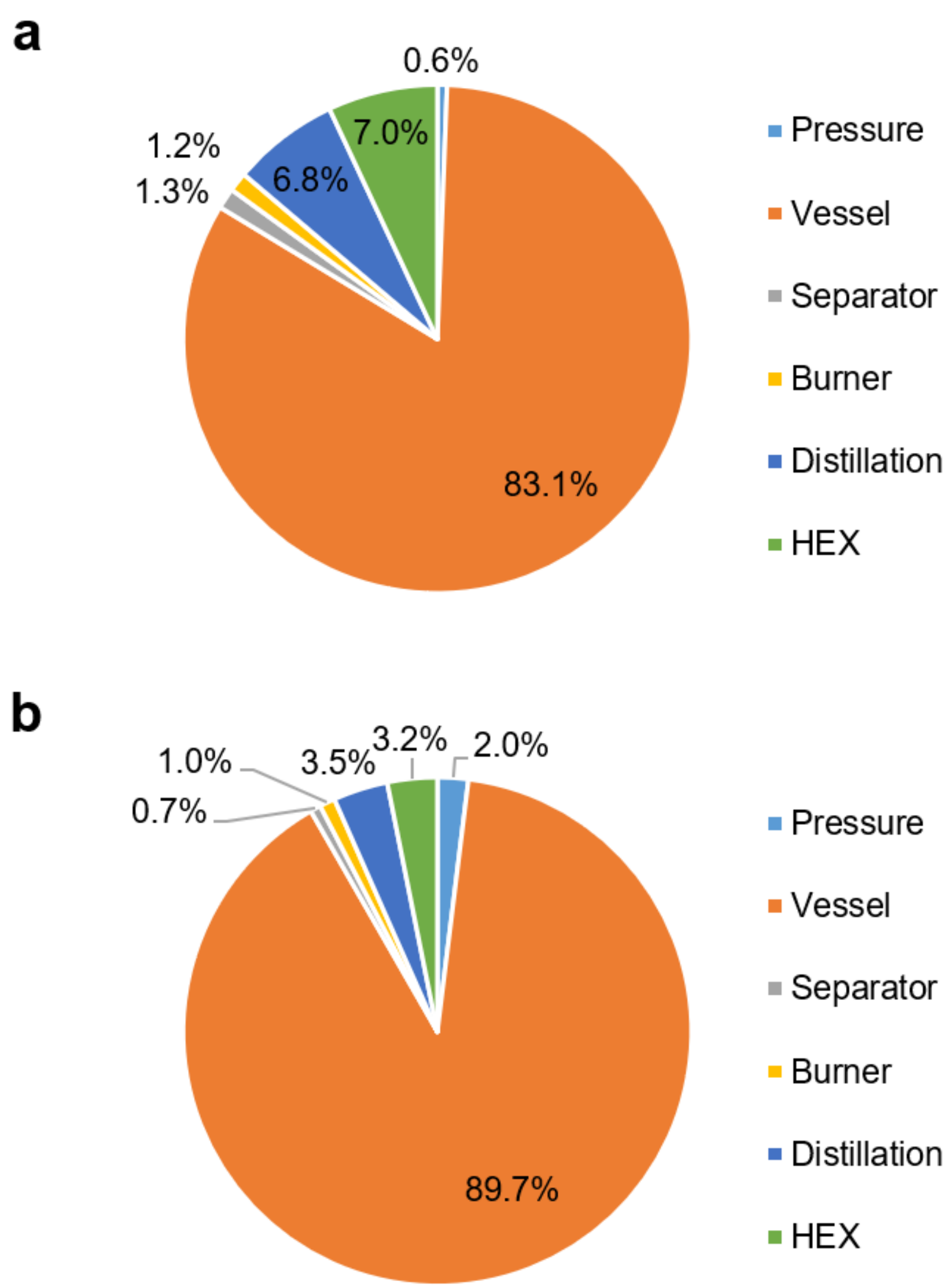

Supplementary Figure 16. CAPEX distribution of (a) MCH-based and (b) H_2-based naphtha production.

3. Supplementary Tables

Supplementary Table 1. Product distribution over different Pt- and Ru-based catalysts in Fig. 1a. Reaction conditions: 2 g PP (Mw∼308 kDa), 0.2 g catalyst, 300 °C, 5 h, 0.02 mol MCH, stirring at 400 RPM, 3 bar He. The mass of MCH addition initially is excluded in the product distribution. All mass balances are within the range of 97% ± 3%.

**Catalyst**	C_1-C_4 (wt%)	C_5-C_8 (wt%)	C_9-C_{12} (wt%)	C_{13+} (wt%)	Solid (wt%)
Pt/Al_2O_3	1.4	18.7	6.1	2.5	71.3
$Pt2Ni/Al_2O_3$	2.2	29.8	10.8	2.5	54.7
$Pt2Co/Al_2O_3$	5.2	38.7	7.1	1.8	47.1
$Pt2Ru/Al_2O_3$	14.4	63.3	10.0	3.5	8.8
Ru/Al_2O_3	3.1	27.6	18.2	3.9	47.3

Supplementary Table 2. Product distribution over PtyRu/Al_2O_3 catalysts with different Ru loadings in Fig. 1b. Reaction conditions: 2 g PP (Mw~308 kDa), 0.2 g catalyst, 300 °C, 5 h, 0.02 mol MCH, stirring at 400 RPM, 3 bar He. The mass of MCH addition initially is excluded in the product distribution. All mass balances are within the range of 97% ± 3%.

**Catalyst**	C_1-C_4 (wt%)	C_5-C_8 (wt%)	C_9-C_{12} (wt%)	C_{13+} (wt%)	Solid (wt%)
Pt/Al_2O_3	1.4	18.7	6.1	2.5	71.3
Pt0.1Ru/Al_2O_3	1.8	25.4	13.0	6.4	53.6
Pt0.5Ru/Al_2O_3	2.0	54.1	8.3	2.9	32.8
PtRu/Al_2O_3	4.5	70.0	18.5	3.2	3.8
Pt2Ru/Al_2O_3	14.4	63.3	10.0	3.5	8.8
Pt5Ru/Al_2O_3	24.0	40.8	14.2	3.2	17.7

Supplementary Table 3. Comparison of the present system with reported LOHC-assisted and hydrogen-driven plastic hydroconversion systems from the literature.

Catalyst	Plastic	Mw (kDa)	Catalyst /Plastic ratio	Temperature (°C)	Time (h)	H_2 pressure (bar)/Solvent amount (g)	C_5-C_{12} production rate (g g-metal⁻^{1} h⁻^{1})	Ref.
$PtRu/Al_2O_3$	PP	308	10	300	5	Methylcyclohexane (1.96)	103.4	This work
$RuPt/TiO_2$	LDPE	4	10	325	24	Methanol (0.2)+ water (1.8)	1.9	[30]
RuPt/C	PE	4	17	300	12	Formic acid (0.18)	2.3	[31]
Pt/ZSM-5	LDPE	4	4	300	10	Decalin (1)	63.2	[32]
PtSnCe/SiAl	HDPE	97	10	270	2	H_2 (30)	116.7	[33]
Rh/Nb_2O_5	LLDPE	-	10	300	6	H_2 (30)	33.9	[34]
Pt/WZr &HY	LDPE	250	10	250	2	H_2 (30)	47.1	[35]
Pt/WZr &BEA	LDPE	250	10	250	2	H_2 (30)	36.8	[35]
Ni/BEA	LDPE	76	100	250	12	H_2 (60)	133.3	[36]
Ru/WZr	LDPE	76	40	250	2	H_2 (50)	28.0	[37]
Ru/VZr	LDPE	76	80	250	2	H_2 (50)	46.0	[37]
Ru/TiZr	LDPE	76	40	250	2	H_2 (50)	18.0	[37]
Ru/ZrO_2	LDPE	34	4	240	4	H_2 (60)	9.0	[38]
Pt/5Ce-HY	LDPE	110	10	300	2	H_2 (30)	66.7	[39]
$RuNiPt/ZrO_2$	LDPE	4	50	250	15	H_2 (20)	31.9	[40]

Supplementary Table 4. Product distribution over $PtRu/Al_2O_3$ catalysts with different temperatures in Fig. 1d. Reaction conditions: 2 g PP (Mw~308 kDa), 0.2 g $PtRu/Al_2O_3$ catalyst, 5 h, 0.02 mol MCH, stirring at 400 RPM, 3 bar He. The mass of MCH addition initially is excluded in the product distribution. All mass balances are within the range of 97% ± 3%.

**Temperature**	C_1-C_4 (wt%)	C_5-C_8 (wt%)	C_9-C_{12} (wt%)	C_{13+} (wt%)	Solid (wt%)
250 °C	1.1	2.2	1.4	0.3	95.0
280 °C	0.7	49.0	6.7	1.9	41.7
300 °C	4.5	70.0	18.5	3.2	3.8

Supplementary Table 5. Product distribution over $PtRu/Al_2O_3$ catalysts with different MCH amount in Fig. 1e. Reaction conditions: 2 g PP (Mw∼308 kDa), 0.2 g $PtRu/Al_2O_3$ catalyst, 300 °C, 5 h, stirring at 400 RPM, 3 bar He. The mass of MCH addition initially is excluded in the product distribution. All mass balances are within the range of 97% ± 3%.

**MCH (mmol)**	C_1-C_4 (wt%)	C_5-C_8 (wt%)	C_9-C_{12} (wt%)	C_{13+} (wt%)	Solid (wt%)
0	2.0	4.4	1.01	0.2	92.5
5	2.3	36.3	13.4	4.2	43.9
10	5.2	50.6	14.4	3.5	26.4
20	4.5	70.0	18.5	3.2	3.8
30	3.1	77.3	13.6	3.4	2.6

Supplementary Table 6. Product distribution over $PtRu/Al_2O_3$ catalysts with different molecular weight of PP in Fig. 1f. Reaction conditions: 2 g PP, 0.2 g $PtRu/Al_2O_3$ catalyst, 300 °C, 0.02 mol MCH, 5 h, stirring at 400 RPM, 3 bar He. The mass of MCH addition initially is excluded in the product distribution. All mass balances are within the range of 97% ± 3%.

**PP Mw (kDa)**	C_1-C_4 (wt%)	C_5-C_8 (wt%)	C_9-C_{12} (wt%)	C_{13+} (wt%)	Solid (wt%)
160	7.4	77.9	10.6	3.7	0.5
167	3.2	72.1	17.7	4.6	2.4
176	5.7	72.7	15.3	3.8	2.5
292	3.8	70.8	17.5	4.5	3.3
308	4.5	70.0	18.5	3.2	3.8

Supplementary Table 7. GPC results for different molecular weight PP.

PP samples	Mw (g/mol)	Mn (g/mol)	Mw / Mn
PP904J	159900	21500	7.4
PP901J	166700	22400	7.5
PP902J	176300	16400	10.8
PP701J	291900	34300	8.5
PP600F	308100	40400	7.6

Supplementary Table 8. ICP-MS results for different Pt-based or Ru-based catalysts.

**Catalyst**	Pt (wt%)	Ru (wt%)	Ni (wt%)	Co (wt%)
Pt/Al_2O_3	0.90			
$Pt0.1Ru/Al_2O_3$	0.89	0.08		
$Pt0.5Ru/Al_2O_3$	0.88	0.40		
$PtRu/Al_2O_3$	0.89	0.85		
$Pt2Ru/Al_2O_3$	0.84	1.69		
$Pt5Ru/Al_2O_3$	0.78	4.52		
Ru/Al_2O_3		0.90		
$Pt2Ni/Al_2O_3$	0.86		1.66	
$Pt2Co/Al_2O_3$	0.83			1.74
$PtRu/Al_2O_3$ (after 5 cycles)	0.86	0.82		

Supplementary Table 9. Product selectivity and solid yield over PtRu/Al_2O_3 catalysts with different reaction time in Fig. 3b. Reaction conditions: 2 g PP, 0.2 g PtRu/Al_2O_3 catalyst, 300 °C, 0.02 mol MCH, stirring at 400 RPM, 3 bar He. The mass of MCH addition initially is excluded in the product distribution. All mass balances are within the range of 97% ± 3%.

Time (h)	C_1-C_4 (wt%)	C_5-C_8 (wt%)	C_9-C_{12} (wt%)	C_{13+} (wt%)	Solid (wt%)
1	2.8	22.5	37.4	37.3	68.3
2	3.8	55.9	24.1	16.2	33.2
3	4.0	60.1	23.9	12.0	18.4
4	4.2	65.3	23.4	7.0	9.5
5	4.7	72.8	19.2	3.3	3.3

Supplementary Table 10. Product distribution over $PtRu/Al_2O_3$ catalysts with different reaction cycles in Fig. 3c. Reaction conditions: 2 g PP, 0.2 g $PtRu/Al_2O_3$ catalyst (only added in the 0th cycle), 300 °C, 0.02 mol MCH, stirring at 400 RPM, 3 bar He. The mass of MCH addition initially is excluded in the product distribution. All mass balances are within the range of 97% ± 3%.

**Recycle number**	C_1-C_4 (wt%)	C_5-C_8 (wt%)	C_9-C_{12} (wt%)	C_{13+} (wt%)	Solid (wt%)
0th	4.5	70.0	18.5	3.2	3.8
1st	4.7	70.3	19.7	2.0	3.3
2nd	4.0	70.8	19.9	2.3	2.4
3rd	4.1	69.8	18.5	2.9	4.7
4th	4.5	70.0	19.0	2.6	4.0
5th	3.6	70.2	19.5	2.9	3.8

Supplementary Table 11. Product distribution over PtRu/Al_2O_3 catalysts with different commercial plastics in Fig. 3d. Reaction conditions: 2 g commercial plastics, 0.2 g PtRu/Al_2O_3 catalyst, 300 °C, 0.02 mol MCH, stirring at 400 RPM, 3 bar He. The mass of MCH addition initially is excluded in the product distribution. All mass balances are within the range of 97% ± 3%.

**Commercial plastic**	C_1-C_4 (wt%)	C_5-C_8 (wt%)	C_9-C_{12} (wt%)	C_{13+} (wt%)	Solid (wt%)
Pipette tips	1.5	71.4	17.3	7.0	2.7
Microtubes	6.9	70.7	16.7	3.0	2.7
Miketea cup	3.7	71.2	20.1	4.1	1.1
Miketea lid	6.9	69.3	13.2	4.2	6.4

Supplementary Table 12. Product distribution over $PtRu/Al_2O_3$ catalysts with different conditions in Fig. 4a. Reaction conditions: 2 g PP, 0.2 g $PtRu/Al_2O_3$ catalyst, 300 °C, 0.02 mol MCH or external hydrogen, 5 h or 10 h, stirring at 400 RPM, 3 bar He. The mass of MCH addition initially is excluded in the product distribution. All mass balances are within the range of 97% ± 3%.

**Reaction conditions**	C_1-C_4 (wt%)	C_5-C_8 (wt%)	C_9-C_{12} (wt%)	C_{13+} (wt%)	Solid (wt%)
MCH + 5 h	4.5	70.0	18.5	3.2	3.8
29 bar H_2 +5 h	9.1	31.8	19.6	8.3	31.2
29 bar H_2 +10 h	12.5	51.8	24.8	6.1	4.8
45 bar H_2 +5 h	24.6	26.7	33.5	9.5	4.9

Supplementary Table 13. GPC results of solid residues over different LOHCs after reaction.

**Solvent**	Mw (g/mol)	Mn (g/mol)	Mw / Mn
MCH	2500	800	3.0
DEC	44200	8400	5.3
Raw PP	308100	40400	7.6

Supplementary Table 14. Product distribution over different solvents in Fig. 4b. Reaction conditions: 2 g PP, 0.2 g PtRu/Al_2O_3 catalyst, 300 °C, 0.02 mol solvent, 5 h, stirring at 400 RPM, 3 bar He. The mass of solvent addition initially is excluded in the product distribution. All mass balances are within the range of 97% ± 3%.

**Solvent**	C_1-C_4 (wt%)	C_5-C_8 (wt%)	C_9-C_{12} (wt%)	C_{13+} (wt%)	Solid (wt%)
MCH	4.5	70.0	18.5	3.2	3.8
CHE	9.7	57.0	10.0	3.6	19.8
DMCH	2.5	37.0	7.9	4.3	31.9
DEC	4.8	0.8	1.7	0.7	92.0
DMP	5.4	17.1	14.7	4.5	58.4
HEP	2.9	37.8	11.6	2.9	44.8
NA	2.0	4.4	1.0	0.2	92.5

Supplementary Table 15. Heat integration network of MCH-based Naphtha production

Load (kJ/h)	Cost ($)	Area (m^2)	U (kJ/h m^2 °C)	Hot stream	Hot in (°C)	Hot out (°C)	Cold stream	Cold in (°C)	Cold out (°C)
1.8E+06	2.5E+04	33.8	683.5	1	300.0	20.0	Cooling Water	10.0	20.0
1.7E+05	1.5E+04	8.7	683.5	2	93.8	20.0	Cooling Water	10.0	20.0
1.9E+04	1.0E+04	0.4	683.5	4	258.0	20.0	Cooling Water	10.0	20.0
2.2E+05	1.6E+04	10.8	683.5	3	93.8	20.0	Cooling Water	10.0	20.0
2.5E+05	1.0E+04	0.4	696.8	Burning	1199.9	1199.9	HPS Generation	249.0	249.1
2.4E+05	1.1E+04	0.7	360.0	Burning	1200.0	1199.9	Reaction	299.9	300.0
2.1E+04	1.0E+04	0.1	360.0	Burning	1199.9	1199.9	Reaction	299.9	299.9
9.3E+03	1.0E+04	0.0	696.8	Burning	1200.0	1200.0	HPS Generation	249.0	249.0
2.6E+06	1.2E+04	4.0	696.8	Burning	1200.0	1199.9	HPS Generation	249.2	250.0
2.4E+05	1.0E+04	0.4	696.8	Burning	1199.9	1199.9	HPS Generation	249.1	249.2

Supplementary Table 16. Heat integration network of H_2-based Naphtha production

Load (kJ/h)	Cost ($)	Area (m^2)	U (kJ/h m^2 °C)	Hot stream	Hot in (°C)	Hot out (°C)	Cold stream	Cold in (°C)	Cold out (°C)
5.7E+04	1.1E+04	1.2	683.5	4	259.4	20.0	Cooling Water	10.0	20.0
8.6E+04	1.2E+04	3.3	683.5	3	127.3	20.0	Cooling Water	10.0	20.0
6.1E+05	1.1E+04	0.9	696.8	Burning	1199.9	1199.9	HPS Generation	249.9	250.0
1.1E+05	1.3E+04	4.4	683.5	2	127.3	20.0	Cooling Water	10.0	20.0
9.0E+05	1.9E+04	16.6	683.5	1	300.0	20.0	Cooling Water	10.0	20.0
1.5E+06	1.6E+04	12.1	696.8	Reaction	300.0	299.9	LPS Generation	124.0	124.9
1.3E+05	1.1E+04	1.1	696.8	Reaction	299.9	299.9	LPS Generation	124.9	125.0
7.0E+06	1.5E+04	10.6	696.8	Burning	1200.0	1199.9	HPS Generation	249.0	249.9

References

1. L. Martínez, R. Andrade, E. G. Birgin and J. M. Martínez, *Journal of Computational Chemistry*, 2009, **30**, 2157–2164.
2. K. Vanommeslaeghe, E. Hatcher, C. Acharya, S. Kundu, S. Zhong, J. Shim, E. Darian, O. Guvench, P. Lopes, I. Vorobyov and A. D. Mackerell Jr., *Journal of Computational Chemistry*, 2010, **31**, 671–690.
3. A. P. Thompson, H. M. Aktulga, R. Berger, D. S. Bolintineanu, W. M. Brown, P. S. Crozier, P. J. in 't Veld, A. Kohlmeyer, S. G. Moore, T. D. Nguyen, R. Shan, M. J. Stevens, J. Tranchida, C. Trott and S. J. Plimpton, *Computer Physics Communications*, 2022, **271**, 108171.
4. T. Darden, D. York and L. Pedersen, *Journal of chemical physics*, 1993, **98**, 10089–10092.
5. J.-P. Ryckaert, G. Ciccotti and H. J. C. Berendsen, *Journal of Computational Physics*, 1977, **23**, 327–341.
6. N. Michaud-Agrawal, E. J. Denning, T. B. Woolf and O. Beckstein, *Journal of Computational Chemistry*, 2011, **32**, 2319–2327.
7. R. J. Gowers, M. Linke, J. Barnoud, T. J. E. Reddy, M. N. Melo, S. L. Seyler, J. Domanski, D. L. Dotson, S. Buchoux and I. M. Kenney, *MDAnalysis: a Python package for the rapid analysis of molecular dynamics simulations*, Report 2575-9752, Los Alamos National Laboratory (LANL), 2019.
8. R. Turton, R. C. Bailie, W. B. Whiting and J. A. Shaeiwitz, *Analysis, synthesis and design of chemical processes*, Pearson education, 2008.
9. S. Jenkins, *Chemical Engineering magazine*, 2023.
10. Z. Zong, N. Koers, G. Cai and D. C. Upham, *Chemical Engineering Journal*, 2024, **487**, 150274.
11. B. Parkinson, M. Tabatabaei, D. C. Upham, B. Ballinger, C. Greig, S. Smart and E. McFarland, *International Journal of Hydrogen Energy*, 2018, **43**, 2540–2555.
12. B. Parkinson, P. Balcombe, J. F. Speirs, A. D. Hawkes and K. Hellgardt, *Energy & Environmental Science*, 2019, **12**, 19–40.
13. Market Price & Insight, https://www.echemi.com, (accessed 03/17, 2026).
14. S. Kaza, L. Yao, P. Bhada-Tata and F. Van Woerden, *What a waste 2.0: a global snapshot of solid waste management to 2050*, World Bank Publications, 2018.
15. F. I. Bashir, R. T. J. Porter, E. Catalanotti and H. Mahgerefteh, *Energies*, 2025, **18**, 2440.
16. IEA, Energy Prices, https://www.iea.org/data-and-statistics/data-product/energy-prices).
17. Tax credits drive carbon capture deployment in our Annual Energy Outlook, https://www.eia.gov/todayinenergy/detail.php?id=65764#, (accessed 03/06, 2026).
18. Municipal Solid Waste Recycling in the United States: Analysis of Current and Alternative Approaches https://nap.nationalacademies.org/catalog/27978/municipal-solid-waste-recycling-in-the-united-states-analysis-of, 20).
19. A. Fivga and I. Dimitriou, *Energy*, 2018, **149**, 865–874.
20. G. Jiang, J. Wang, S. M. Al-Salem and G. A. Leeke, *Energy & Fuels*, 2020, **34**, 7397–7409.

21. F. Riedewald, Y. Patel, E. Wilson, S. Santos and M. Sousa-Gallagher, *Waste Management*, 2021, **120**, 698–707.
22. W. Leal Filho, J. Barbir, E. Carpio-Vallejo, A. Dobri and V. Voronova, *Science of The Total Environment*, 2025, **999**, 180337.
23. M. Bachmann, C. Zibunas, J. Hartmann, V. Tulus, S. Suh, G. Guillén-Gosálbez and A. Bardow, *Nature Sustainability*, 2023, **6**, 599–610.
24. G. Wernet, C. Bauer, B. Steubing, J. Reinhard, E. Moreno-Ruiz and B. Weidema, *The International Journal of Life Cycle Assessment*, 2016, **21**, 1218–1230.
25. OECD, Plastic waste by region and end-of-life fate, https://data-explorer.oecd.org/vis?tenant=archive&df[ds]=DisseminateArchiveDMZ&df[id]=DF_PLASTIC_WASTE_5&df[ag]=OECD&dq=.&lom=LASTNPERIODS&lo=5&to[TIME_PERIOD]=false&vw=ov, (accessed 03/06, 2026).
26. OECD, Greenhouse gas emissions from plastics lifecycle, https://data-explorer.oecd.org/vis?tenant=archive&df[ds]=DisseminateArchiveDMZ&df[id]=DF_PLASTIC_GHG_2&df[ag]=OECD&dq=.&lom=LASTNPERIODS&lo=5&to[TIME_PERIOD]=false&vw=ov, (accessed 03/06, 2026).
27. Eurostat, *Journal*, 2025.
28. E. Parliament, Plastic waste and recycling in the EU: facts and figures, https://www.europarl.europa.eu/topics/en/article/20181212STO21610/plastic-waste-and-recycling-in-the-eu-facts-and-figures).
29. Energy Statistics Database | United Nations Statistics Division, https://data.un.org/Data.aspx?d=EDATA&f=cmID%3ANP).
30. J. Lee, T. Kwon, K. Hyuk Kang, W. Won and I. Ro, *Angewandte Chemie International Edition*, 2025, **64**, e202420748.
31. Y. Wang, Q. Hu, S. Qian, J. Zhao, Y. Cheng, J. Ma, J. Zhang and Z. Niu, *Nature Communications*, 2025, **16**, 7821.
32. T. Kwon, S. Ahn, T. Kim, J. H. Kang, S. Jeong, K. H. Kang, W. Won and I. Ro, *Nature Communications*, 2025, **17**, 918.
33. H. Wang, T. Yoskamtorn, J. Zheng, P.-L. Ho, B. Ng and S. C. E. Tsang, *ACS Catalysis*, 2023, **13**, 15886–15898.
34. B. Du, X. Chen, Y. Ling, T. Niu, W. Guan, J. Meng, H. Hu, C.-W. Tsang and C. Liang, *ChemSusChem*, 2023, **16**, e202202035.
35. S. Liu, P. A. Kots, B. C. Vance, A. Danielson and D. G. Vlachos, *Science Advances*, 2021, **7**, eabf8283.
36. B. C. Vance, Z. Yuliu, S. Najmi, E. Selvam, J. E. Granite, K. Yu, M. G. Ierapetritou and D. G. Vlachos, *Chemical Engineering Journal*, 2024, **487**, 150468.
37. C. Wang, T. Xie, P. A. Kots, B. C. Vance, K. Yu, P. Kumar, J. Fu, S. Liu, G. Tsilomelekis, E. A. Stach, W. Zheng and D. G. Vlachos, *JACS Au*, 2021, **1**, 1422–1434.

38. M. Tamura, S. Miyaoka, Y. Nakaji, M. Tanji, S. Kumagai, Y. Nakagawa, T. Yoshioka and K. Tomishige, *Applied Catalysis B: Environmental*, 2022, **318**, 121870.
39. X. Wu, X. Wang, L. Zhang, X. Wang, S. Song and H. Zhang, *Angewandte Chemie International Edition*, 2024, **63**, e202317594.
40. D. Li, R. Tsuzuki and S. Furukawa, *JACS Au*, 2026, **6**, 2480–2488.
